# Single-neuron projectome-guided analysis reveals the neural circuit mechanism underlying endogenous opioid antinociception

**DOI:** 10.1093/nsr/nwae195

**Published:** 2024-06-04

**Authors:** Yan-Nong Dou, Yuan Liu, Wen-Qun Ding, Qing Li, Hua Zhou, Ling Li, Meng-Ting Zhao, Zheng-Yi-Qi Li, Jing Yuan, Xiao-Fei Wang, Wang-Yuan Zou, Anan Li, Yan-Gang Sun

**Affiliations:** Institute of Neuroscience, Key Laboratory of Brain Cognition and Brain-Inspired Intelligence Technology, CAS Center for Excellence in Brain Science & Intelligence Technology, Chinese Academy of Sciences, Shanghai 200031, China; Institute of Neuroscience, Key Laboratory of Brain Cognition and Brain-Inspired Intelligence Technology, CAS Center for Excellence in Brain Science & Intelligence Technology, Chinese Academy of Sciences, Shanghai 200031, China; Department of Biology, School of Life Science and Technology, ShanghaiTech University, Shanghai 201210, China; Lingang Laboratory, Shanghai 200031, China; Institute of Neuroscience, Key Laboratory of Brain Cognition and Brain-Inspired Intelligence Technology, CAS Center for Excellence in Brain Science & Intelligence Technology, Chinese Academy of Sciences, Shanghai 200031, China; University of Chinese Academy of Sciences, Beijing 100049, China; Institute of Neuroscience, Key Laboratory of Brain Cognition and Brain-Inspired Intelligence Technology, CAS Center for Excellence in Brain Science & Intelligence Technology, Chinese Academy of Sciences, Shanghai 200031, China; Institute of Neuroscience, Key Laboratory of Brain Cognition and Brain-Inspired Intelligence Technology, CAS Center for Excellence in Brain Science & Intelligence Technology, Chinese Academy of Sciences, Shanghai 200031, China; Institute of Neuroscience, Key Laboratory of Brain Cognition and Brain-Inspired Intelligence Technology, CAS Center for Excellence in Brain Science & Intelligence Technology, Chinese Academy of Sciences, Shanghai 200031, China; University of Chinese Academy of Sciences, Beijing 100049, China; Britton Chance Center for Biomedical Photonics, Wuhan National Laboratory for Optoelectronics, MoE Key Laboratory for Biomedical Photonics, Huazhong University of Science and Technology, Wuhan 430074, China; Department of Anesthesiology, Xiangya Hospital, Central South University, Changsha 410008, China; Britton Chance Center for Biomedical Photonics, Wuhan National Laboratory for Optoelectronics, MoE Key Laboratory for Biomedical Photonics, Huazhong University of Science and Technology, Wuhan 430074, China; HUST-Suzhou Institute for Brainsmatics, JITRI, Suzhou 215123, China; Institute of Neuroscience, Key Laboratory of Brain Cognition and Brain-Inspired Intelligence Technology, CAS Center for Excellence in Brain Science & Intelligence Technology, Chinese Academy of Sciences, Shanghai 200031, China; Department of Anesthesiology, Xiangya Hospital, Central South University, Changsha 410008, China; Britton Chance Center for Biomedical Photonics, Wuhan National Laboratory for Optoelectronics, MoE Key Laboratory for Biomedical Photonics, Huazhong University of Science and Technology, Wuhan 430074, China; HUST-Suzhou Institute for Brainsmatics, JITRI, Suzhou 215123, China; Institute of Neuroscience, Key Laboratory of Brain Cognition and Brain-Inspired Intelligence Technology, CAS Center for Excellence in Brain Science & Intelligence Technology, Chinese Academy of Sciences, Shanghai 200031, China

**Keywords:** endogenous opioid antinociception, mu-opioid receptors, central amygdala nucleus, parabrachial nucleus, single-neuron projectome

## Abstract

Endogenous opioid antinociception is a self-regulatory mechanism that reduces chronic pain, but its underlying circuit mechanism remains largely unknown. Here, we showed that endogenous opioid antinociception required the activation of mu-opioid receptors (MORs) in GABAergic neurons of the central amygdala nucleus (CEA) in a persistent-hyperalgesia mouse model. Pharmacogenetic suppression of these CEA^MOR^ neurons, which mimics the effect of MOR activation, alleviated the persistent hyperalgesia. Furthermore, single-neuron projection analysis revealed multiple projectome-based subtypes of CEA^MOR^ neurons, each innervating distinct target brain regions. We found that the suppression of axon branches projecting to the parabrachial nucleus (PB) of one subtype of CEA^MOR^ neurons alleviated persistent hyperalgesia, indicating a subtype- and axonal-branch-specific mechanism of action. Further electrophysiological analysis revealed that suppression of a distinct CEA-PB disinhibitory circuit controlled endogenous opioid antinociception. Thus, this study identified the central neural circuit that underlies endogenous opioid antinociception, providing new insight into the endogenous pain modulatory mechanisms.

## Teaser

Complex nano-geometries are always accompanied by different local-environments. This work reveales that geometry variations dominate the local strain change, resulting in a shift in the electrochemical reduction reaction path of CO2.

## INTRODUCTION

Opioids have been used for pain relief for thousands of years and are the most powerful painkillers. Opioid receptors and their ligands are widely expressed in the pain pathway, indicating the potential role of endogenously produced opioid peptides in modulating pain processing [1,2]. The analgesic effect of endogenous opioids has been demonstrated in many pharmacological studies [3–5]. Recent studies have also shown that neurons involved in endogenous opioid analgesia in inflammatory pain are distinct from those involved in exogenous opioids [6]. Thus, in addition to the use of opioid-related analgesic drugs, enhancing endogenous opioid analgesia by targeting endogenous opioid release or the underlying circuits could be an alternative approach to pain management. In this study, we aimed to elucidate the neural circuit mechanism underlying endogenous opioid antinociception, a type of endogenous analgesia that contributes to the resolution of hyperalgesia and is mediated by the mu-opioid receptor (MOR), which is known to play a dominant role in exogenous and endogenous opioid analgesia [6–8].

Human brain imaging studies have demonstrated MOR activation in cortical and subcortical brain regions during sustained muscle pain [9,10], indicating that a broad neural network is involved in endogenous opioid analgesia. Consistent with this finding, pharmacological studies on animals showed that MOR activation in many brain areas could contribute to endogenous opioid analgesia [5,11,12]. Additionally, the opioid system in the spinal cord is implicated in endogenous opioid analgesia during inflammatory pain, and pain insensitivity in humans carrying mutations in Na_V_1.7 [13,14]. Recent studies have begun to explore how MOR activation leads to endogenous opioid analgesia. Genetic deletion experiments in mice showed that MOR activation in supraspinal GABAergic neurons is required [6,7]. However, the site of action of endogenous opioids remains unclear.

High-level MOR expression was found in brain regions dominated by GABAergic neurons, such as the striatum, nucleus accumbens (ACB) and central amygdala nucleus (CEA) [6]. Among these regions, the CEA is known to be an important brain region for pain modulation [15]. Recent studies have also demonstrated that distinct subsets of CEA neurons play pronociceptive or antinociceptive roles in pain modulation [16,17]. Moreover, some CEA interneurons are enkephalinergic [18,19], and could participate in endogenous analgesia via MOR in the CEA. However, it remains unclear whether MOR activation in the CEA occurs during endogenous opioid antinociception and how MOR-expressing CEA neurons (CEA^MOR^ neurons) regulate the neural circuit underlying endogenous opioid antinociception.

The axons of CEA neurons project to multiple brain regions, most of which are pain- and emotion-related [20,21]. However, the projection pattern at the single-neuron level, such as whether a single CEA neuron targets one or multiple brain regions, remains to be clarified. This information on the whole-brain projection pattern of a single neuron (‘projectome’) is critical for understanding the functional role of a neuron within the neural circuit. Recent studies have started to map single-neuron projectomes in mice and have revealed projectome-defined subtypes, each with distinct target preferences [22–25]. Thus, mapping single-neuron projectomes of MOR-expressing neurons could not only help to reveal the complexity of CEA neuron connectivity but also guide the study of circuit mechanisms underlying endogenous opioid analgesia.

In the present study, we used a variety of experimental approaches, including region-specific genetic manipulation, pharmacogenetic and optogenetic manipulations, electrophysiological slice recording, and single-neuron projectome analysis, to determine the neural circuit basis of endogenous opioid antinociception, a type of endogenous analgesia that contributes to the resolution of hyperalgesia. We have identified a specific amygdala to the parabrachial nucleus (PB) circuit, in which MOR activation by endogenous opioids results in the suppression of CEA^MOR^ neurons, leading to the inhibition of PB excitatory neurons via a di-synaptic disinhibitory circuit. Given that the PB represents the critical relay nucleus that processes chronic pain [26], this CEA-PB pathway could represent the core circuit that regulates persistent hyperalgesia via endogenous opioids. Our study also illustrates the use of single-neuron projectome analysis to determine the neural circuit underlying a specific brain function.

## RESULTS

### MORs expressed in the CEA are crucial for endogenous opioid antinociception

**Figure 1.**
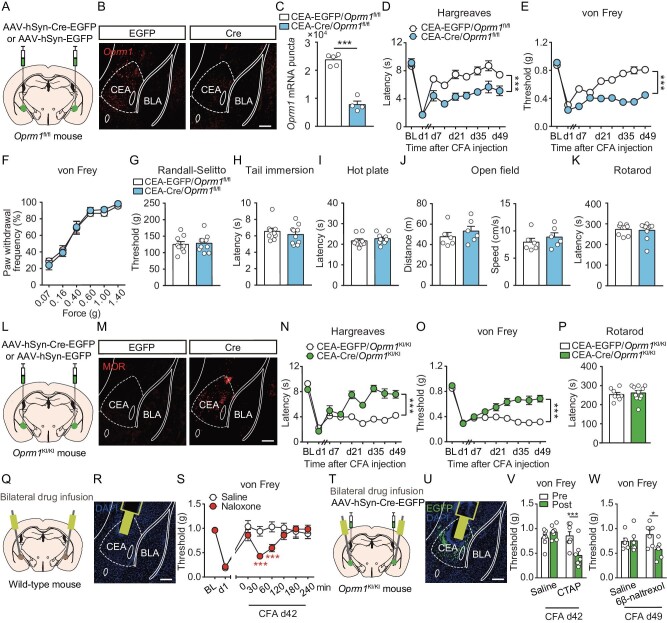
MORs expressed in CEA are crucial for endogenous opioid antinociception. (A) Schematic showing bilateral injection of AAV-hSyn-Cre-EGFP or AAV-hSyn-EGFP virus into the CEA of a male *Oprm1*^fl/fl^ mouse. (B) *In situ* hybridization showing the *Oprm1* expression in CEA of example mice. Scale bar, 200 μm. (C) Quantification of *Oprm1* mRNA puncta in the CEA of the two groups of mice. *n* = 4–5 mice. Student's unpaired *t* test. (D and E) Thermal (D) and mechanical (E) hyperalgesia on CFA-induced persistent hyperalgesia in ipsilateral hindpaws of CEA-EGFP/*Oprm1*^fl/fl^ and CEA-Cre/*Oprm1*^fl/fl^ mice. *n* = 13 mice for each group. BL, baseline, indicating the basal nociceptive sensitivity before CFA injection. d1, day 1. Two-way ANOVA followed by Bonferroni correction. (F and G) Effects of *Oprm1* deletion in the CEA on mechanical nociception tested with von Frey (F) and Randall-Selitto (G) tests. *n* = 9–10 mice. Two-way ANOVA followed by Bonferroni correction (F) and Student's unpaired *t* test (G). (H and I) Effects of *Oprm1* deletion in the CEA on thermal nociception tested with tail immersion (48°C, H) and hot plate (52°C, I) tests. *n* = 9–10 mice. Student's unpaired *t* test. (J) Effects of *Oprm1* deletion in the CEA on locomotor activity in the open field test. *n* = 6 mice for each group. Student's unpaired *t* test. (K) Effects of *Oprm1* deletion in the CEA on motor ability in the rotarod test. *n* = 9–10 mice. Student's unpaired *t* test. (L) Schematic showing bilateral injection of AAV-hSyn-Cre-EGFP or AAV-hSyn-EGFP virus into the CEA of a male *Oprm1*^KI/KI^ mouse. (M) Immunostaining showing the MOR re-expression in the CEA of an *Oprm1*^KI/KI^ mouse. Scale bar, 200 μm. (N and O) Thermal (N) and mechanical (O) hyperalgesia on CFA-induced persistent hyperalgesia in ipsilateral hindpaws of *Oprm1*^KI/KI^ mice. *n* = 17–19 mice. BL, baseline, indicating the basal nociceptive sensitivity before CFA injection. Two-way ANOVA followed by Bonferroni correction. (P) Effects of MOR re-expression in the CEA on motor ability in the rotarod test. *n* = 8–11 mice. Student's unpaired *t* test. (Q) Schematic showing bilateral implantation of cannulas in the CEA of a male wild-type mouse. (R) Graph showing the track of the cannula in the CEA. Scale bar, 200 μm. (S) Effects of a competitive opioid receptor antagonist (naloxone, 200 ng/0.5 μl) on nociceptive threshold on day 42 (d42) after CFA application, when hyperalgesia was mostly recovered, in wild-type mice. *n* = 7 mice for each group. BL, baseline, indicating the basal nociceptive sensitivity before CFA injection. Two-way ANOVA followed by Bonferroni correction. (T) Schematic showing bilateral virus injection and cannula implantation in the CEA of a male *Oprm1*^KI/KI^ mouse. (U) Graph showing the virus expression and cannula track in the CEA. Scale bar, 200 μm. (V and W) Effects of MOR-specific neutral antagonists CTAP (300 ng/0.5 μl) and 6β-Naltrexol, (1 μg/0.5 μl) on nociceptive threshold on day 42 (d42) and d49, respectively, after CFA application, when hyperalgesia was mostly recovered, in *Oprm1*^KI/KI^ mice. *n* = 6–7 mice. Two-way ANOVA followed by Bonferroni correction. *P < 0.05, ***P < 0.001. Data are presented as mean ± SEM. See also Fig. S1.

Given that MOR in the amygdala is activated by sustained pain conditions [10], we hypothesized that MORs expressed in CEA neurons, which provide major amygdala output to other brain regions, mediate the antinociceptive effect of endogenous opioids. To test this hypothesis, we selectively deleted *Oprm1*, which encodes MOR, from CEA neurons by injecting an adeno-associated virus (AAV) encoding Cre recombinase (AAV-hSyn-Cre-EGFP), or AAV-hSyn-EGFP as a control, into the bilateral CEA of *Oprm1*^fl/fl^ mice (Fig. 1A and Fig. S1A–D). These mice are referred to hereafter as CEA-Cre/*Oprm1*^fl/fl^ and CEA-EGFP/*Oprm1*^fl/fl^ mice, respectively. We examined the efficiency of MOR deletion with RNAscope, and found that *Oprm1* mRNA was significantly reduced in the CEA of CEA-Cre/*Oprm1*^fl/fl^ mice, compared with CEA-EGFP/*Oprm1*^fl/fl^ mice (Fig. 1B and C), whereas the mRNA levels of three other genes were not significantly affected (Fig. S1E and F). Thus, MORs were selectively deleted in the CEA of CEA-Cre/*Oprm1*^fl/fl^ mice. The functional role of MORs in the CEA in endogenous opioid antinociception was further examined by monitoring the recovery from complete Freund's adjuvant (CFA)-induced persistent hyperalgesia, since endogenous opioid antinociception is known to be essential for this recovery [6,13]. In CEA-EGFP/*Oprm1*^fl/fl^ mice, we found that the paw withdrawal latency (in the Hargreaves test) and mechanical nociceptive threshold (in the von Frey test) decreased to a low level immediately after intraplantar CFA injection, indicating CFA-induced hyperalgesia, and gradually recovered to near baseline levels 3 weeks after intraplantar CFA injection, which was consistent with previous reports [6,13]. In contrast, CEA-Cre/*Oprm1*^fl/fl^ mice with MOR deletion in the CEA exhibited sustained hyperalgesia (Fig. 1D and E). Nociceptive behaviors, locomotion and motor coordination were not significantly affected by selective *Oprm1* deletion in the CEA (Fig. 1F–K). Thus, genetic deletion of MORs in CEA slowed recovery from CFA-induced persistent hyperalgesia, suggesting the loss of endogenous opioid antinociception.

The notion that CEA MORs are essential for endogenous opioid antinociception was further supported by the finding that selectively re-expressing MORs in the CEA in MOR-knockout mice restored endogenous opioid antinociception. We used *Oprm1*^KI/KI^ mice, which were generated by inserting one stop cassette flanked by two *loxP* sites between exon 1 and exon 2 of the *Oprm1* gene, leading to the blockade of MOR expression and loss of endogenous opioid antinociception [6]. This mouse line enables selective re-expression of MORs in distinct neuronal populations under the control of Cre recombinase. To re-express MOR in CEA neurons, we injected AAV-hSyn-Cre-EGFP virus or AAV-hSyn-EGFP virus as a control into the bilateral CEA of *Oprm1*^KI/KI^ mice (Fig. 1L). These mice are hereafter referred to as CEA-Cre/*Oprm1*^KI/KI^ and CEA-EGFP/*Oprm1*^KI/KI^ mice, respectively. CEA-Cre/*Oprm1*^KI/KI^ mice showed selective expression of MORs in the CEA (Fig. 1M) and exhibited gradual recovery from CFA-induced persistent hyperalgesia (Fig. 1N and O). In contrast, control CEA-EGFP/*Oprm1*^KI/KI^ mice exhibited persistent hyperalgesia after CFA administration (Fig. 1N and O). Similar results were obtained in female mice using the same strategy (Fig. S1M–P). On the other hand, re-expression of MORs in the CEA did not significantly affect nociceptive behaviors, locomotion or motor coordination (Fig. 1P, Fig. S1G–L). Thus, endogenous antinociception was restored in MOR knockout mice by selective re-expression of MORs in CEA neurons, indicating the requirement of MORs in CEA for endogenous opioid antinociception.

Next, we examined whether MOR activation in CEA neurons during persistent hyperalgesia was mediated by opioids released endogenously in the CEA or by agonist-independent constitutive MOR activation. In mice recovering from CFA-induced persistent hyperalgesia, the administration of a MOR antagonist effectively reverses the analgesic effects mediated by endogenous opioids [27]. We thus locally infused a competitive opioid receptor antagonist (naloxone, 200 ng/0.5 μl) into the bilateral CEA of wild-type mice, after the mice recovered from CFA-induced persistent hyperalgesia (Fig. 1Q–S). We found that the mechanical nociceptive threshold decreased after the administration of naloxone (Fig. 1S), indicating the reinstatement of persistent hyperalgesia. Local drug infusion does not differentiate between the potential effects of MORs on CEA neurons and those on presynaptic axon projections from other brain areas. Thus, we further examined the effects of MOR-specific neutral antagonists (CTAP, 300 ng/0.5 μl and 6β-naltrexol, 1 μg/0.5 μl) on CFA-induced persistent hyperalgesia in CEA-Cre/*Oprm1*^KI/KI^ mice, in which MORs were only expressed in CEA neurons but not presynaptic axon terminals from other brain regions (Fig. 1T and U). We found that these antagonists had comparable effects on reinstating persistent hyperalgesia in CEA-Cre/*Oprm1*^KI/KI^ mice (Fig. 1V and W), indicating that antinociception is mediated by MOR activation in CEA neurons. Furthermore, since the neutral antagonist could not block agonist-independent constitutive MOR activation [13,28], these results indicate that MOR activation in CEA was mediated by endogenously released opioids. Thus, these results indicate that tonic release of endogenous opioids is responsible for analgesia by activating MORs in CEA neurons during persistent hyperalgesia.

### Increased excitability of CEA^MOR^ neurons during CFA-induced persistent hyperalgesia

**Figure 2.**
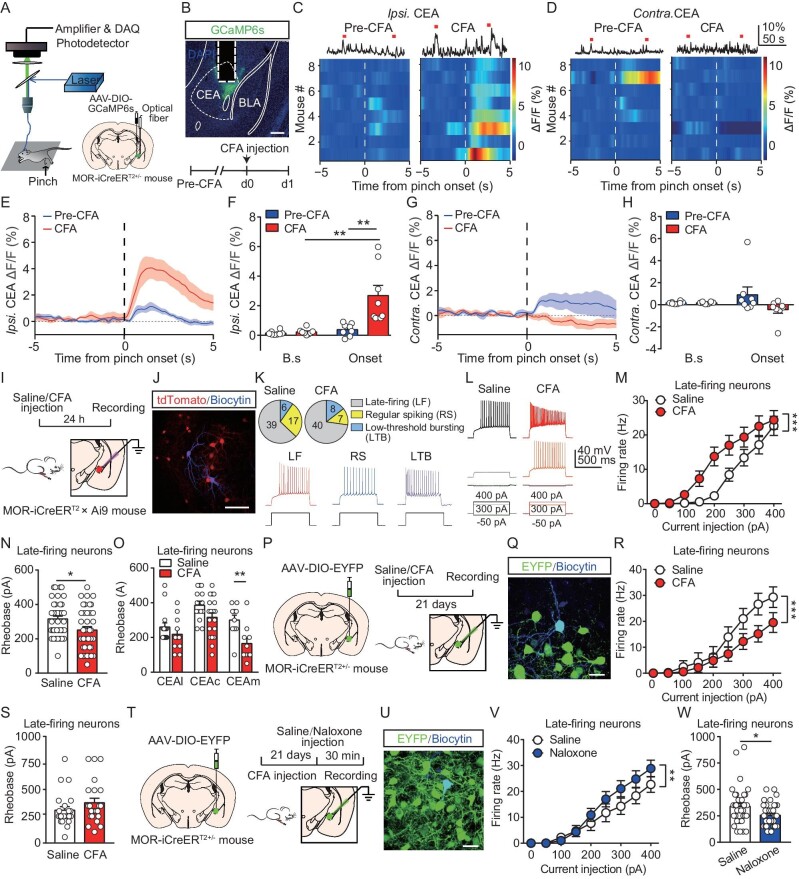
The excitability of CEA^MOR^ neurons increased during persistent hyperalgesia. (A) Schematic diagram showing the recording system for fiber photometry and stereotaxic injection into the right CEA. (B) Graph showing the AAV-DIO-GCaMP6s expression in a male MOR-iCreER^T2+/-^ mouse and dashed line outlining the optical fiber (top). Scale bar, 200 μm. Schematic diagram and timeline of the experiment (bottom). (C and D) Representative photometry traces (top) and averaged GCaMP6s fluorescence dynamics for each mouse relative to the pinch onset (bottom) before (Pre-CFA, left) and 1 day after CFA (CFA, right) application in response to ipsilateral (*Ipsi.*, C) and contralateral (*Contra.*, D) hindpaw pinch. Each row represents the response of one mouse. (E–H) Time course of calcium activity in response to *Ipsi.* (E and F) and *Contra.* (G and H) hindpaw pinch and comparison of the averaged fluorescence signal change during paw pinch baseline (-5–0 s, B.s) and onset period (0–5 s) in each session. *n* = 8 mice for each group. Two-way ANOVA followed by Bonferroni correction. (I) Schematic showing the electrophysiological recording on tdTomato-labeled MOR^+^ neurons in the ipsilateral CEA of a male MOR-iCreER^T2^×Ai9 mouse, which was injected with CFA (50%, 20 μl) or saline 1 day before. (J) *Post hoc* staining of a recorded tdTomato-labeled CEA^MOR^ neuron with biocytin. Scale bar, 50 μm. (K) The percentage of the recorded CEA^MOR^ neurons with different firing patterns (top) and showcase of firing patterns (bottom). Saline group: *n* = 62 neurons from 3 mice; CFA group: *n* = 55 neurons from 5 mice. (L–N) Representative traces of currents evoked by current injection (L), data of firing rates (M) and rheobase values (N) for neurons with ‘late-firing’ pattern. Saline group: *n* = 39 neurons from 3 mice; CFA group: *n* = 40 neurons from 5 mice. Student's unpaired *t* test (N), and two-way ANOVA followed by Bonferroni correction (M). (O) Rheobase for neurons with ‘late-firing’ pattern in CEA sub-regions. Saline group, *n* = 15 (CEAl), 14 (CEAc), 10 (CEAm) neurons from 3 mice; CFA group: *n* = 11 (CEAl), 19 (CEAc), 10 (CEAm) neurons from 5 mice. Two-way ANOVA followed by Bonferroni correction. (P) Schematic showing the electrophysiological recording on EYFP-labeled MOR^+^ neurons in the ipsilateral CEA of a male MOR-iCreER^T2+/−^ mouse, which was injected with CFA (50%, 20 μl) or saline 21 days before. (Q) *Post hoc* staining of a recorded EYFP-labeled CEA^MOR^ neuron with biocytin. Scale bar, 20 μm. (R and S) Data of firing rates (R) and rheobase values (S) in neurons with ‘late-firing’ pattern. *n* = 23 neurons from 4 mice for each group. Student's unpaired *t*- test (S), and two-way ANOVA followed by Bonferroni correction (R). (T) Schematic showing the electrophysiological recording on EYFP-labeled MOR^+^ neurons in the ipsilateral CEA of a male MOR-iCreER^T2+/−^ mouse, which was injected with CFA (50%, 20 μl) 21 days before. Mice were perfused for collection of a brain slice section for recording 30 min after naloxone (3 mg/kg, intraperitoneal, i.p.) or saline injection. (U) *Post hoc* staining of a recorded EYFP-labeled CEA^MOR^ neuron with biocytin. Scale bar, 20 μm. (V and W) Data of firing rates (V) and rheobase values (W) in neurons with ‘late-firing’ pattern. *n* = 35–38 neurons from 3 mice for each group. Student's unpaired *t*- test (W), and two-way ANOVA followed by Bonferroni correction (V). * *P* < 0.05, ** *P* < 0.01, *** *P* < 0.001. Data are presented as mean ± SEM. See also Figs S2 and S3, Movies S1 and S2.

The involvement of CEA^MOR^ neurons, which are widely distributed in the lateral (CEAl), capsular (CEAc) and medial (CEAm) subdivisions of the amygdala (Fig. S2, Movies S1 and S2), in the inflammatory hyperalgesia was further examined by measuring the activity of CEA^MOR^ neurons with *in vivo* fiber photometry. The fluorescent Ca^2+^ probe GCaMP6s was expressed specifically in CEA^MOR^ neurons by injecting AAV-hSyn-DIO-GCaMP6s into the right CEA of MOR-iCreER^T2+/−^ mice, and optical fibers were implanted above the injection site (Fig. 2A and B). One month after viral injection, the response of CEA^MOR^ neurons in the right CEA to right but not left hindpaw pinch (noxious mechanical stimulus) was markedly increased after intraplantar CFA injection compared with the responses in the same group of mice before CFA treatment (Fig. 2C–H). We further analyzed the location of the tip of optical fibers in the CEA subregions, and found that the signal we collected was mainly from the population in the CEAm and CEAl (Fig. S3A and B). These results indicate that the excitability of a specific group of CEA^MOR^ neurons was greatly increased during persistent hyperalgesia induced by CFA injection, consistent with that observed during chronic pain induced by nerve injury [17].

The excitability of CEA^MOR^ neurons during CFA-induced persistent hyperalgesia was further examined by whole-cell patch clamp recording in brain slices. CEA neurons are known to exhibit different firing patterns [17]. We obtained recordings from tdTomato-labeled MOR neurons in the right CEA of MOR-iCreER^T2^×Ai9 mice after intraplantar injection of saline or CFA in the right hindpaw (Fig. 2I and J). We found that CEA^MOR^ neurons exhibited three stereotypical firing patterns following step-depolarization: ‘late-firing’ neurons that began spiking ∼400 ms after current injection (Fig. S3C and D), ‘regular-spiking’ neurons showing steady spiking, and ‘low-threshold bursting’ neurons exhibiting a brief burst of spiking followed by steady spiking. ‘Late-firing’ neurons were predominant, and their percentages in the CEA were comparable between saline- and CFA-treated mice (Fig. 2K, Fig. S3F and G). Notably, we found that ‘late-firing’ CEA^MOR^ neurons in CFA-treated mice exhibited a significantly increased firing rate (Fig. 2L and M) and reduced rheobase (Fig. 2N) compared to those of saline-treated mice. We found that most of these rheobase-reduced neurons were located in the CEAm but not in the CEAl or CEAc (Fig. 2O). The resting membrane potential (RMP) was similar between two groups of mice (Fig. S3E). In contrast, the physiological properties of CEA^MOR^ neurons with two other firing patterns did not exhibit significant differences between the two groups (Fig. S3H–S). Thus, the intrinsic excitability of the ‘late-firing’ CEA^MOR^ neurons was selectively increased in CFA-induced persistent hyperalgesia.

To further investigate the involvement of ‘late-firing’ CEA^MOR^ neurons in CFA-induced persistent hyperalgesia, we measured the excitability of CEA^MOR^ neurons 3 weeks after CFA injection when the mice had recovered from persistent hyperalgesia (Fig. 2P and Q). We found that the firing rate of CEA^MOR^ neurons in the CFA-injected group was significantly decreased (Fig. 2R), and there was no significant difference in the rheobase (Fig. 2S) compared to that of saline-treated mice. We next examined whether activation of the opioid system is involved in the recovery of excitability in CEA^MOR^ neurons. We determined the effect of naloxone, an antagonist of opioid receptors, on neuronal excitability on day 21 after CFA injection (Fig. 2T and U). We found that the firing rate was significantly increased (Fig. 2V) and the rheobase was markedly decreased (Fig. 2W) in ‘late-firing’ EYFP-labeled CEA^MOR^ neurons after naloxone treatment compared with that in saline-treated mice, which suggests that the recovery of excitability in CEA^MOR^ neurons is mediated by activation of the opioid system. Consistently, we found that the activation of MORs by bath application of the MOR agonist DAMGO during slice recording markedly reduced the excitability of these ‘late-firing’ CEA^MOR^ neurons (Fig. S3T–W). Taken together, these results suggest that MOR activation is involved in the reverse of increased excitability of ‘late-firing’ CEA^MOR^ neurons after recovery of hyperalgesia.

### Effects of bidirectional manipulation of CEA^MOR^ neuronal activity

**Figure 3.**
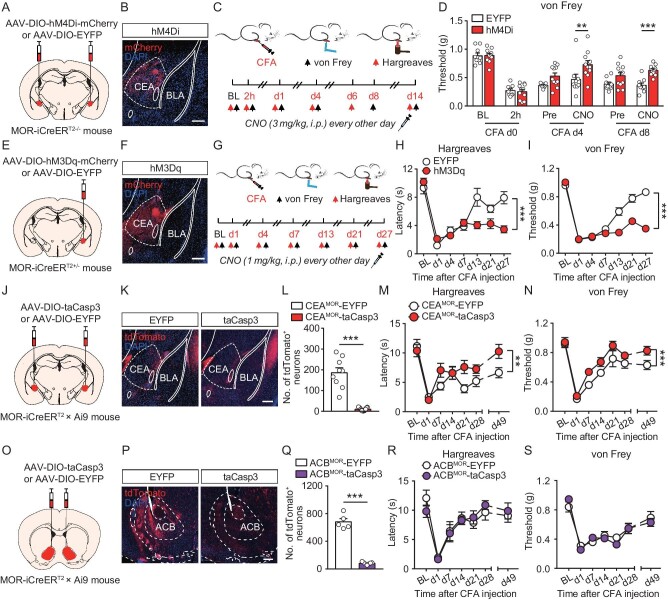
Effect of bidirectional manipulation of CEA^MOR^ neuronal activity on nociception modulation. (A) Schematic showing bilateral injection of AAV-DIO-hM4Di-mCherry or AAV-DIO-EYFP virus into the CEA of a male MOR-iCreER^T2-/−^ mouse. (B) Representative expression of AAV-DIO-hM4Di-mCherry virus in the CEA of MOR-iCreER^T2-/−^ mouse. Scale bar, 200 μm. (C) Schematic diagram showing design of the experiment and timeline of the behavioral tests in CFA-induced persistent hyperalgesia. BL, baseline, indicating the basal nociceptive sensitivity before CFA injection. d1, day 1. (D) Mechanical hyperalgesia on CFA-induced persistent hyperalgesia in ipsilateral hindpaws of MOR-iCreER^T2-/−^ mice before (Pre) and 30 min after CNO (3 mg/kg, i.p.) injection on specific days after CFA injection. *n* = 9–11 mice. BL, baseline, indicating the basal nociceptive sensitivity before CFA injection. Two-way ANOVA followed by Bonferroni correction. (E) Schematic showing injection of AAV-DIO-hM3Dq-mCherry or AAV-DIO-EYFP virus into the right CEA of a male MOR-iCreER^T2+/−^ mouse. (F) Graph showing representative expression of AAV-DIO-hM3Dq-mCherry virus in the CEA. Scale bar, 200 μm. (G) Schematic diagram showing design of the experiment and timeline of the behavioral tests in CFA-induced persistent hyperalgesia. BL, baseline, indicating the basal nociceptive sensitivity before CFA injection. (H and I) Thermal (H) and mechanical (I) hyperalgesia on CFA-induced persistent hyperalgesia in ipsilateral hindpaws of male MOR-iCreER^T2+/−^ mice after CNO (1 mg/kg, i.p.) injection every other day. *n* = 7–10 mice. BL, baseline, indicating the basal nociceptive sensitivity before CFA injection. Two-way ANOVA followed by Bonferroni correction. (J) Schematic showing bilateral injection of AAV-hSyn-DIO-taCasp3 or AAV-hSyn-DIO-EYFP virus into the CEA of a MOR-iCreER^T2^×Ai9 mouse. (K) Distribution of tdTomato^+^ neurons in the CEA of MOR-iCreER^T2^×Ai9 mice, which were injected with AAV-hSyn-DIO-taCasp3 or AAV-hSyn-DIO-EYFP virus into the bilateral CEA. Scale bar, 200 μm. (L) Total number of tdTomato^+^ neurons in the CEA of a MOR-iCreER^T2^×Ai9 mouse. *n* = 9–17 mice. Student's unpaired *t* test. (M and N) Thermal (M) and mechanical (N) hyperalgesia on CFA-induced persistent hyperalgesia in ipsilateral hindpaws of a male MOR-iCreER^T2^×Ai9 mouse. *n* = 5–10 mice. BL, baseline, indicating the basal nociceptive sensitivity before CFA injection. Two-way ANOVA followed by Bonferroni correction. (O) Schematic showing bilateral injection of AAV-hSyn-DIO-taCasp3 or AAV-hSyn-DIO-EYFP virus into the ACB of a male MOR-iCreER^T2^×Ai9 mouse. (P) Distribution of tdTomato^+^ neurons in the ACB of MOR-iCreER^T2^×Ai9 mice, which were injected with AAV-hSyn-DIO-taCasp3 or AAV-hSyn-DIO-EYFP virus into the bilateral ACB. Scale bar, 200 μm. (Q) Total number of tdTomato^+^ neurons in the ACB of a MOR-iCreER^T2^×Ai9 mouse. *n* = 5–7 mice. Student's unpaired *t* test. (R and S) Thermal (R) and mechanical (S) hyperalgesia on CFA-induced persistent hyperalgesia in ipsilateral hindpaws of a MOR-iCreER^T2^×Ai9 mouse. *n* = 5–7 mice. BL, baseline, indicating the basal nociceptive sensitivity before CFA injection. Two-way ANOVA followed by Bonferroni correction. **P < 0.01, ***P < 0.001. Data are presented as mean ± SEM. See also Fig. S4.

It is likely that the increase in the excitability of CEA^MOR^ neurons gates CFA-induced persistent hyperalgesia. If this were true, the suppression of CEA^MOR^ neurons should mimic the antinociceptive effect of MOR activation due to endogenous opioids and reduce CFA-induced persistent hyperalgesia. To test this hypothesis, we repetitively suppressed the activity of CEA^MOR^ neurons by a pharmacogenetic approach in mice lacking MORs. We stereotaxically injected the Cre-dependent AAV virus encoding hM4Di, the inhibitory designer receptor that is exclusively activated by designer drugs (DREADDs) [29] or AAV-Ef1α-DIO-EYFP virus as a control, bilaterally into the CEA of MOR-iCreER^T2-/−^ mice (Fig. 3A and B, and Fig. S4B–E), in which MORs were deleted (Fig. S4A). In CFA-treated mice, pharmacogenetic inhibition of CEA^MOR^ neurons by intraperitoneal injection of clozapine-N-oxide (CNO, 3 mg/kg) every other day significantly decreased persistent hyperalgesia, as indicated by the increase in the mechanical nociceptive threshold and paw withdrawal latency (Fig. 3C and D, Fig. S4F and G), but there were no effects on the locomotion or motor coordination of the mice (Fig. S4H and I). Conversely, we examined whether prolonged activation of CEA^MOR^ neurons could aggravate persistent hyperalgesia. We expressed the excitatory DREADD receptor hM3Dq in CEA^MOR^ neurons in MOR-iCreER^T2+/−^ mice and found that intraperitoneal injection of CNO (1 mg/kg) increased CEA^MOR^ neuronal responses to right hindpaw pinch (Fig. S4J–O) to a level comparable to that in mice after intraplantar CFA injection (Fig. 2F). Furthermore, we pharmacogenetically activated CEA^MOR^ neurons by intraperitoneal injection of CNO (1 mg/kg) every other day from CFA d0 to d20 in MOR-iCreER^T2+/−^ mice injected with AAV-DIO-hM3Dq-mCherry or AAV-DIO-EYFP into the right CEA (Fig. 3E–G). We found that prolonged activation of CEA^MOR^ neurons aggravated CFA-induced persistent hyperalgesia without significantly affecting locomotion (Fig. 3G–I, Fig. S4P and Q). These results suggest that the increased excitability of CEA^MOR^ neurons gates CFA-induced persistent hyperalgesia.

We next examined the crucial role of CEA^MOR^ neurons in endogenous analgesia by bilateral ablation of CEA^MOR^ neurons with a caspase3-based method [30]. We bilaterally injected AAV-CAG-DIO-taCasp3-TEVp virus, or AAV-Ef1α-DIO-EYFP virus as a control, into the CEA of MOR-iCreER^T2^×Ai9 mice (Fig. 3J), yielding CEA^MOR^-taCasp3 and CEA^MOR^-EYFP mice, respectively. We found that the number of CEA^MOR^ neurons labeled with tdTomato in the CEA of CEA^MOR^-taCasp3 mice was significantly lower than that in CEA^MOR^-EYFP control mice (Fig. 3K and L). This finding was also confirmed by counting *Oprm1*^+^ neurons with RNAscope (Fig. S4R–T). The ablation of MOR neurons was specific to the CEA, since the number of *Oprm1*^+^ neurons in two nearby regions, the dorsal and ventral endopiriform nucleus, was not significantly affected (Fig. S4T and U). By measuring the thermal nociceptive latency and mechanical nociceptive threshold, we found that the recovery of CFA-induced persistent hyperalgesia was faster in CEA^MOR^-taCasp3 mice than in control mice (Fig. 3M and N), confirming the key role of CEA^MOR^ neurons in endogenous opioid antinociception. Notably, ablation of CEA^MOR^ neurons did not significantly affect nociception induced by thermal or mechanical stimuli and locomotion in the absence of CFA treatment (Fig. S4V–Y). These CEA neurons are known to be GABAergic [6]; thus, this result is consistent with previous studies showing that MORs in GABAergic neurons are involved in endogenous opioid analgesia [6,7]. In contrast, we found that the ablation of MOR-expressing neurons in the ACB, which is enriched with GABAergic neurons, did not affect CFA-induced persistent hyperalgesia (Fig. 3O–S), which is consistent with a previous study showing intact opioid analgesia in mice with GABAergic MOR knockout in the forebrain [31]. These results further support the specific role of CEA^MOR^ neurons in endogenous opioid antinociception. Taken together, these data indicate that CEA^MOR^ neurons are essential for endogenous opioid antinociception and that their activity directly gates persistent hyperalgesia induced by CFA.

### Single-cell projectome of CEA^MOR^ neurons reveals projection-based subtypes

**Figure 4.**
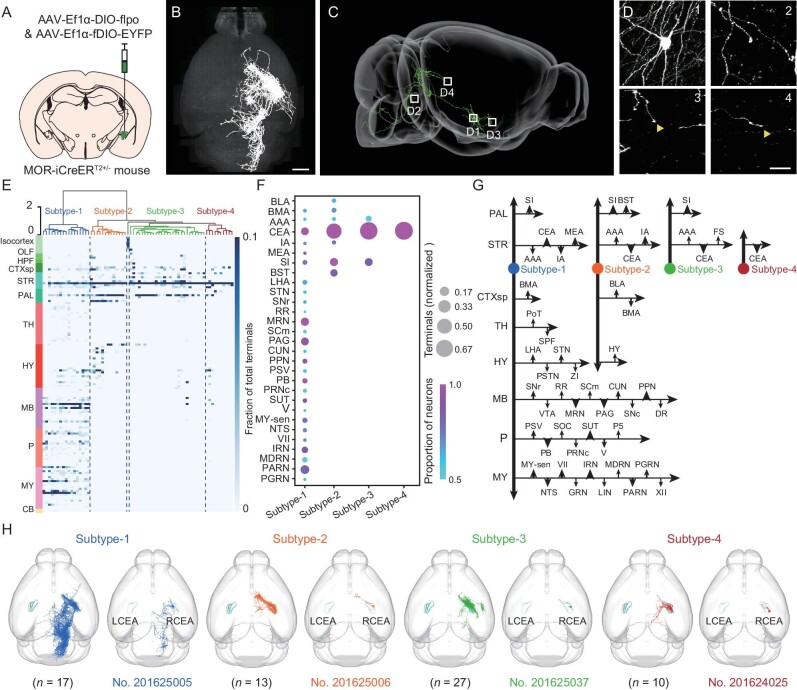
Single-cell projectome of CEA^MOR^ neurons reveals projection-based subtypes. (A) Schematic depiction of sparse labeling strategy by injection of sparse labeling virus into the right CEA of a male MOR-iCreER^T2+/−^ mouse. (B) Horizontal view of whole-brain imaging of an example sparsely labeled MOR-iCreER^T2+/−^ mouse brain. Scale bar, 1 mm. (C) Graph showing a representative CEA^MOR^ neuron in sagittal view. (D) Raw data of 83 × 83 × 83 μm^3^ containing the soma (D1 in (C)) or terminals (arrows, D2–D4 in (C)) of the example neuron (D1 in (C)). Scale bar, 20 μm. (E) Clustering of CEA^MOR^ neurons based on their whole-brain projectome. The dendrogram was color-coded according to the subtypes division. Heat map colors represent projection strength (number of terminals) normalized by total number of terminals for each neuron. Target sub-regions were grouped and colored according to the Allen Reference Atlas. (F) Primary target regions of each subtype. Colors represent the proportion of neurons that projected to the region in each subtype. The size of the circles represents the averaged number of terminals that was normalized by total number of terminals. For each subtype, only regions that receive projection from >50% neurons in this subtype were plotted. (G) A summary wiring diagram of CEA^MOR^ neurons. Abbreviations for brain regions are listed in Table S1. (H) Horizontal views demonstrating the overviews of the reconstructed CEA^MOR^ neurons. A total of 67 CEA^MOR^ neurons clustered by 4 groups, total neurons (left) and single neuron (right) in each group, were reconstructed with their target regions in the mouse brain. Different colors correspond to different subtypes of neurons. *n* = 67 neurons from 4 mice. The neuron counts ranged from 9 to 31 for these 4 mice. See also Figs S5 and S6, and Table S1.

CEA neurons are the principal output neurons of the amygdala and have multiple downstream target brain regions [21]. Viral tracing by expressing a fluorescent protein in CEA^MOR^ neurons showed that these neurons sent axon projections to multiple downstream targets (Fig. S5). The projection pattern can determine the function of different neuronal subtypes within the same brain region [32]. To elucidate the circuit underlying the function of CEA^MOR^ neurons in endogenous opioid antinociception and how these neurons gate persistent hyperalgesia, we performed single-neuron projectome analysis to comprehensively classify these neurons based on their projection patterns. We mapped the single-neuron projectome of CEA^MOR^ neurons from four mouse brain samples. The neurons in the right CEA of MOR-iCreER^T2+/−^ mice were EYFP-labeled with a recently developed sparse labeling approach [33] (Fig. 4A–D), and whole-brain data sets of EYFP-labeled CEA^MOR^ neurons and axonal and dendritic processes were acquired using high-definition fluorescence micro-optical sectioning tomography (HD-fMOST) [34]. The brain-wide axon projections of 67 individual CEA^MOR^ neurons were reconstructed by a semiautomatic tracing method [22] and registered to the Allen Mouse Brain Common Coordinate Framework (CCFv3) [35]. We determined the projection strength of each neuron by estimating the total axon arbor length within the target area and the percentage of CEA^MOR^ neurons projecting to that area [22]. We found strong projections of CEA^MOR^ neurons to the midbrain reticular nucleus (MRN), lateral hypothalamic area (LHA), PB and bed nuclei of the stria terminalis (BST) (Fig. S6A and B).

To classify CEA^MOR^ neurons based on their axon projection patterns, we used NBLAST to measure pairwise neuronal similarity [36]. Four different subtypes of CEA^MOR^ neurons were identified based on their projections by hierarchical clustering using Euclidean distance metric and Ward's linkages (Fig. 4E–H and Fig. S6C), excluding subtypes with fewer than 10 reconstructed neurons. Subtype-1 CEA^MOR^ neurons exhibited the most extensive axons with primarily posterior long-range projections to mid- and hindbrain areas, including the PB, MRN and periaqueductal gray (PAG) (Fig. 4E–G). Subtype-2 neurons primarily projected to the BST and substantia innominate (SI), whereas subtype-3 and subtype-4 CEA^MOR^ neurons projected to forebrain regions such as the cortical subplate (CTXsp), striatum (STR) and pallidum (PAL) (Fig. 4E–G). Notably, this projectome analysis revealed that each CEA^MOR^ neuron of the same subtype projected axons to multiple target areas with distinct neuron-specific target preferences (Fig. 4E). Moreover, we found that the somata of each CEA^MOR^ subtype were spatially clustered in CEA subregions (Fig. S6D–G). The somata of subtype-1 were clustered in the CEAl and CEAm. However, subtype-2 to subtype-4 neurons were distributed primarily in the CEAc (Fig. S6D–G). Subtype-1 neurons also exhibited marked differences in other projection characteristics, such as axon length, number of stems, number of bifurcations, number of branches and number of endcounts, compared with the other subtypes, indicating a more complex projection pattern (Fig. S6H–L). Thus, distinct functions may be served by CEA^MOR^ neurons of different projection subtypes as well as by different axon branches of the sameneuron.

### The amygdala-PB pathway mediates the antinociceptive effect of CEA^MOR^ neurons

**Figure 5.**
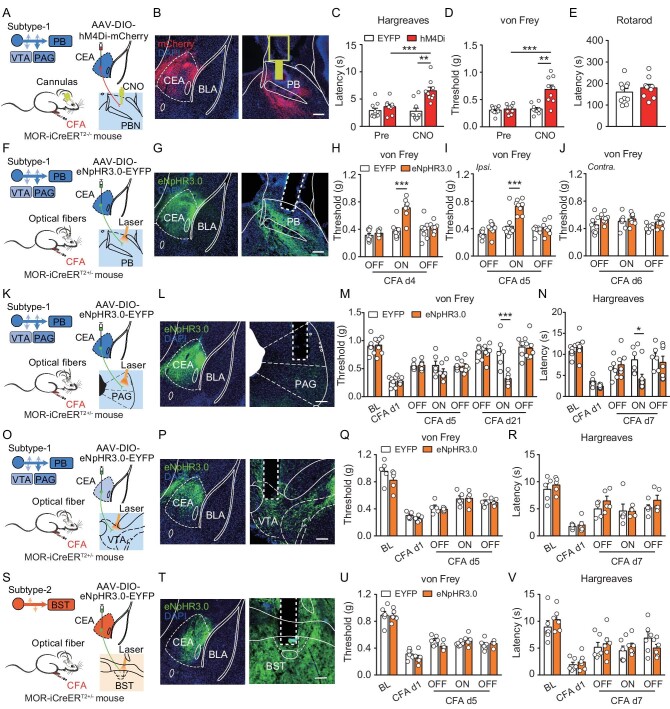
CEA^MOR^-PB circuit underlying the modulation of endogenous antinociception. (A) Schematic showing injection of AAV-DIO-hM4Di-mCherry virus into the right CEA and implantation of a cannula above the right PB of a male MOR-iCreER^T2-/−^ mouse, which was induced with persistent hyperalgesia by injection of CFA in the right hindpaw. (B) Immunostaining showing representative expression of mCherry^+^ neurons in the CEA and mCherry^+^ fibers in PB of a MOR-iCreER^T2-/−^ mouse. Scale bar, 200 μm. (C and D) Effects of pharmacogenetic suppression of the ipsilateral projection from subtype-1 CEA^MOR^ neurons to the PB on thermal (C) and mechanical (D) hyperalgesia on CFA-induced persistent hyperalgesia in ipsilateral hindpaws of MOR-iCreER^T2-/−^ mice before (Pre) and 30 min after CNO (0.5 μg/0.5 μl) infusion in the PB on day 6 after CFA-application. *n* = 9–10 mice. BL, nociceptive sensitivity tested before CNO injection. Two-way ANOVA followed by Bonferroni correction. (E) Effects of pharmacogenetic suppression of the ipsilateral projection from subtype-1 CEA^MOR^ neurons to the PB on motor ability in the rotarod test. *n* = 9–10 mice. Student's unpaired *t* test. (F) Schematic showing bilateral injection of AAV-DIO-eNpHR3.0-EYFP virus into the CEA, and implantation of optical fibers above the PB of a male MOR-iCreER^T2+/−^ mouse. (G) Graph showing representative expression of EYFP^+^ neurons in the CEA and EYFP^+^ fibers in the PB. Scale bar, 200 μm. (H–J) Effects of optogenetic suppression of the ipsilateral projection from subtype-1 CEA^MOR^ neurons to the PB on nociceptive threshold after CFA-induced mechanical hyperalgesia on specific days after CFA application ((H), bilateral suppression on CFA day (d) 4; (I), ipsilateral inhibition suppression on CFA d5; (J), contralateral suppression on CFA d6). *n* = 8–9 mice. Two-way ANOVA followed by Bonferroni correction. (K) Schematic showing injection of AAV-Ef1α-DIO-eNpHR3.0-EYFP virus into the right CEA, and implantation of optical fiber above the right PAG of a male MOR-iCreER^T2+/−^ mouse. (L) Immunostaining showing representative expression of EYFP^+^ neurons in the right CEA and EYFP^+^ fibers in the right PAG. Scale bar, 200 μm. (M and N) Effects of optogenetic suppression of the ipsilateral projection from subtype-1 CEA^MOR^ neurons to PAG on mechanical (M) and thermal (N) hyperalgesia on CFA-induced persistent hyperalgesia in ipsilateral hindpaws on specific days after CFA application. *n* = 6–7 mice. BL, baseline, indicating the basal nociceptive sensitivity before CFA injection. d1, day 1. Two-way ANOVA followed by Bonferroni correction. (O) Schematic showing injection of AAV-Ef1α-DIO-eNpHR3.0-EYFP virus into the right CEA, and implantation of optical fiber above the right VTA of a male MOR-iCreER^T2+/−^ mouse. (P) Immunostaining showing representative expression of EYFP^+^ neurons in the right CEA and EYFP^+^ fibers in the right VTA. Scale bar, 200 μm. (Q and R) Effects of optogenetic suppression of the ipsilateral projection from subtype-1 CEA^MOR^ neurons to VTA on mechanical (Q) and thermal (R) hyperalgesia on CFA-induced persistent hyperalgesia in ipsilateral hindpaws on specific days after CFA application. *n* = 5 mice for each group. BL, baseline, indicating the basal nociceptive sensitivity before CFA injection. Two-way ANOVA followed by Bonferroni correction. (S) Schematic showing injection of AAV-Ef1α-DIO-eNpHR3.0-EYFP virus into the right CEA, and implantation of optical fiber above the right BST of a male MOR-iCreER^T2+/−^ mouse. (T) Immunostaining showing representative expression of EYFP^+^ neurons in the right CEA and EYFP^+^ fibers in the right BST. Scale bar, 200 μm. (U and V) Effects of optogenetic suppression of the ipsilateral projection from subtype-2 CEA^MOR^ neurons to BST on mechanical (U) and thermal (V) hyperalgesia on CFA-induced persistent hyperalgesia in ipsilateral hindpaws on specific days after CFA application. *n* = 6 mice for each group. BL, baseline, indicating the basal nociceptive sensitivity before CFA injection. Two-way ANOVA followed by Bonferroni correction. *P < 0.05, **P < 0.01, ***P < 0.001. Data are presented as mean ± SEM. See also Figs S7 and S8.

Single-neuron projectome analysis of CEA^MOR^ neurons provided the basis for deciphering the circuit mechanism underlying the role of CEA^MOR^ neurons in endogenous opioid antinociception. We found that all CEA^MOR^ neurons in projection subtype-1 projected to the PB, a key relay brain region for nociceptive information processing [26,37]. Using whole-cell recording of brain slices, we confirmed that CEA^MOR^ neurons provided inhibitory inputs to PB neurons (Fig. S7A–G), which was consistent with previous reports [38,39]. We then tested the involvement of the pathway from subtype-1 CEA^MOR^ neurons to the PB in endogenous opioid antinociception by determining whether suppressing this pathway mimicked the effect of MOR activation in the CEA to alleviate CFA-induced persistent hyperalgesia. To selectively suppress the synaptic terminals of CEA^MOR^ neurons in the PB, we injected the AAV-hSyn-DIO-hM4Di-mCherry virus, or AAV-Ef1α-DIO-EYFP virus as a control, into the right CEA of MOR-iCreER^T2-/−^ mice and implanted a cannula above the ipsilateral PB for CNO infusion (Fig. 5A and B). We found that CFA-induced persistent hyperalgesia was alleviated by local ipsilateral CNO infusion in hM4Di-expressing mice, in which ipsilateral projections from CEA^MOR^ neurons to PB were suppressed, and the same CNO infusion in EYFP-expressing control mice had no effect (Fig. 5C and D). In contrast, CFA-induced persistent hyperalgesia was not affected by saline infusion in either group (Fig. S7H–K). Moreover, suppressing CEA^MOR^ neuron projections to the PB did not affect the motor functions of mice (Fig. 5E). The importance of CEA-to-PB projections for endogenous antinociception was further confirmed with an optogenetic approach, using virally infected mice expressing eNpHR3.0 in CEA^MOR^ neurons (Fig. 5F and G). We found that optogenetic suppression of bilateral or ipsilateral but not contralateral projections from subtype-1 CEA^MOR^ neurons to the PB markedly alleviated CFA-induced persistent hyperalgesia (Fig. 5H–J), without affecting basal nociceptive sensitivity or locomotion (Fig. S7L–O). Previous studies have shown that neurons expressing protein kinase C-delta (PKCδ^+^ neurons) in the CEA were antinociceptive in a model of nerve injury [17,18]. We found that *Oprm1*^+^ neurons only partially overlapped with *Prkcd*^+^ and somatostatin-expressing (*Sst*^+^) neurons (Fig. S8A–D). Moreover, we found that *Prkcd* was expressed in the CEAc and CEAl, while EYFP-labeled PB-projecting CEA^MOR^ neurons were distributed in the CEAm. Thus, PB-projecting CEA^MOR^ neurons are distinct from PKCδ^+^ neurons in the CEA (Fig. S8E–G).

Since subtype-1 CEA^MOR^ neurons projected to multiple brain areas, we further examined whether collateral axon branches projecting to other brain areas besides the PB contributed to modulation of nociception. Our projectome data indicated that the vast majority of subtype-1 CEA^MOR^ neurons projected to the ipsilateral PAG (Fig. 4F and G, and Fig. S6M), which represents a crucial component of the descending pain modulatory pathway and receives GABAergic input from the CEA [2,40–44]. We examined the effect of optogenetic suppression of the axonal terminals of subtype-1 CEA^MOR^ neurons in the PAG (Fig. 5K and L), and found that this suppression induced a trend of decrease in the mechanical nociceptive threshold on CFA d5 (Fig. 5M). One of the possible reasons could be that CFA induced hyperalgesia in the early stage, leading to the limited effect of aggravation. Since we found that the same suppression induced hyperalgesia, as evidenced by a decrease in the paw withdrawal latency and mechanical nociceptive threshold in response to basal nociceptive sensitivity (Fig. S7P and Q) without significantly affecting locomotion (Fig. S7R), the effect of optogenetic suppression of the projection on CFA-induced hyperalgesia was further determined on CFA d7 and d21, when the hyperalgesia was gradually recovered (Fig. 5M and N). We found that suppression of the CEA^MOR^-PAG projection induced a significant decrease in the mechanical nociceptive threshold and paw withdrawal latency (Fig. 5M and N), indicating that this suppression induced hyperalgesia. Additionally, half of subtype-1 CEA^MOR^ neurons projected to the ipsilateral ventral tegmental area (VTA) (Fig. 4F and G, and Fig. S6M), which is known to be involved in pain modulation [45]. We next examined the effect of optogenetically suppressing subtype-1 CEA^MOR^ neuron projections in the VTA (Fig. 5O and P), and found that this suppression did not significantly affect the nociceptive threshold during CFA-induced persistent hyperalgesia and locomotion (Fig. 5Q and R, and Fig. S7S–U). This result suggests that PB-projecting axon branches of subtype-1 CEA^MOR^ neurons play an essential role in endogenous opioid antinociception.

We also examined the role of another projection subtype of CEA^MOR^ neurons in endogenous opioid antinociception, particularly subtype-2 neurons that projected to the BST (Fig. 5S and T), which is known to be involved in pain processing [46,47]. We found that optogenetic suppression of CEA^MOR^ axon projections in the BST, which represents a major target of subtype-2 neurons (Fig. 4F and G, and Fig. S6M), had no effect on CFA-induced persistent hyperalgesia and locomotion (Fig. 5U and V, and Fig. S7V–X), indicating the projection subtype specificity of CEA^MOR^ neurons in the endogenous modulation of nociception. Taken together, these data demonstrate that projection subtype-1 CEA^MOR^ neurons modulate CFA-induced persistent hyperalgesia via their projections to the PB, and that this modulation is target- and projection-subtype specific.

### The CEA^MOR^-PB circuit underlying endogenous antinociception

**Figure 6.**
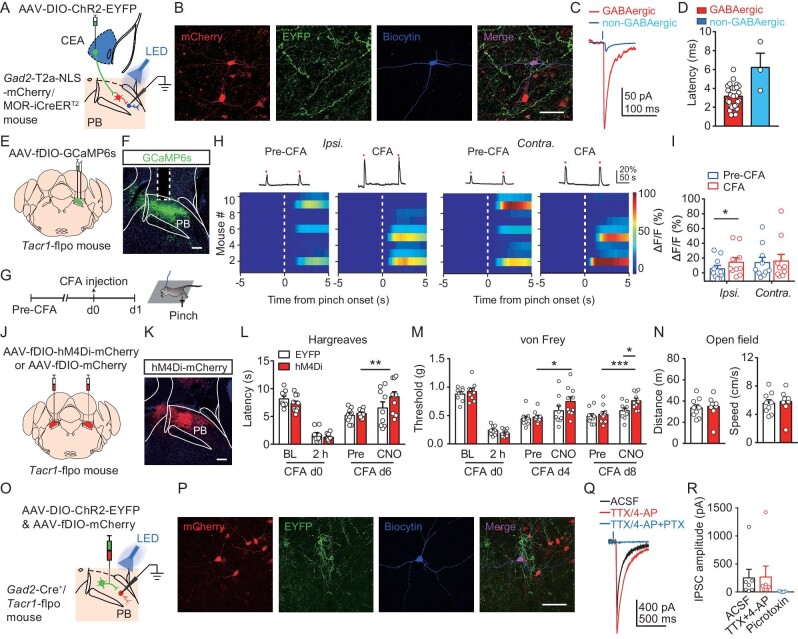
A di-synaptic disinhibitory local circuit in the PB involved in the modulation of nociception by CEA^MOR^-PB projection. (A) Schematic showing the injection of AAV-DIO-ChR2-EYFP into the right CEA of a male *Gad2*-T2a-NLS-mCherry/MOR-CreER^T2^ mouse followed by patch clamp recording of mCherry^+^ (GABAergic) and mCherry^−^ (non-GABAergic) neurons in the right PB. (B) *Post hoc* staining of recorded GABAergic with biocytin and EYFP^+^ fibers. Scale bar, 50 μm. (C) Representative light-evoked IPSCs recorded from GABAergic and non-GABAergic PB neurons. Blue bar, LED stimulation (475 nm; 1 ms). (D) Averaged latency of light-evoked IPSCs recorded in PB neurons. *n* = 3–28 neurons from 9 mice. (E) Schematic showing injection of AAV-hSyn-fDIO-GCaMP6s virus into the right PB, and implantation of optical fiber above the right PB of a male *Tacr1*-flpo mouse. (F) Representative image showing the virus expression and optical fiber tip location in the PB. Scale bar, 200 μm. (G) Schematic showing the design of the experiment. d0, day 0. (H) Averaged GCaMP6s fluorescence dynamics relative to the pinch onset before (Pre-CFA) and 1 day after CFA application in response to ipsilateral (*Ipsi.*, left) and contralateral (*Contra.*, right) hindpaw pinch. (I) Comparison of the averaged fluorescence signal change during paw pinch onset period (0–5 s) in each session. *n* = 10 mice. Student's paired *t* test. (J) Schematic showing bilateral injection of AAV-Ef1α-fDIO-hM4Di-mCherry or AAV-Ef1α-fDIO-mCherry virus into the PB of a male *Tacr1*-flpo mouse. (K) Representative expression of AAV-Ef1α-fDIO-hM4Di-mCherry virus in the PB of a *Tacr1*-flpo mouse. Scale bar, 200 μm. (L and M) Thermal (L) and mechanical (M) hyperalgesia on CFA-induced persistent hyperalgesia in ipsilateral hindpaws of *Tacr1*-flpo mice before (Pre) and 30 min after CNO (3 mg/kg, i.p.) injection on specific days after CFA injection. *n* = 9–11 mice. BL, baseline, indicating the basal nociceptive sensitivity before CFA injection. Two-way ANOVA followed by Bonferroni correction. (N) Effects of pharmacogenetic suppression of PB*^Tacr1^* neurons on locomotor activity in the open field test. *n* = 10 mice for each group. Student's unpaired *t* test. (O) Schematic showing injection of mixed AAV-DIO-ChR2-EYFP and AAV-fDIO-mCherry viruses into the PB of a male *Gad2*-Cre^+^/*Tacr1*-flpo mouse followed by patch clamp recording of mCherry^+^ (PB*^Tacr1^* neurons) in the PB. (P) *Post hoc* staining of a recorded mCherry labeled *Tacr1^+^* neuron with biocytin and EYFP^+^ fibers. Scale bar, 50 μm. (Q) Representative light-evoked IPSCs blocked by picrotoxin (PTX, 50 μM) recorded from PB*^Tacr1^* neurons. Bar, LED stimulation (475 nm; 1 ms). (R) Summary of the IPSCs evoked by local GABAergic inputs before and after PTX application. *n* = 7 neurons from 3 mice. *P < 0.05, **P < 0.01, ***P < 0.001. Data are presented as mean ± SEM. See also Fig. S9.

Next, we investigated the local circuit involved in the endogenous modulation of nociception. Activation of glutamatergic neurons in the PB is known to induce pain-related behaviors [48]. We hypothesized that an increase in the activity of CEA^MOR^ neurons during persistent hyperalgesia may increase the activity of PB glutamatergic neurons via CEA^MOR^-PB projections; thus, suppressing the activity of CEA^MOR^ neurons by endogenous opioids could reduce persistent hyperalgesia. Given that CEA^MOR^ neurons are GABAergic [6], we hypothesized that activation of the CEA^MOR^-PB pathway increases the activity of PB excitatory neurons via a local disinhibitory circuit in the PB. We thus examined the synaptic connectivity between CEA^MOR^ neurons and GABAergic and non-GABAergic neurons in the PB. We generated MOR-iCreER^T2^/*Gad2*-T2a-NLS-mCherry mice, in which ChR2 and mCherry could be expressed in CEA^MOR^ neurons and GABAergic neurons, respectively, via local viral injection (Fig. 6A and B, see Methods). Whole-cell patch-clamp recording was performed on brain slices to measure inhibitory postsynaptic currents (IPSCs) from GABAergic (mCherry^+^) and non-GABAergic (mCherry^−^) neurons in the PB (Fig. 6A–C). We found that 46.7% (28/60) of PB GABAergic neurons exhibited induced IPSCs in response to focal PB photostimulation of MOR^+^ fibers originating from the CEA. In contrast, only 11.5% (3/26) of non-GABAergic neurons in the PB exhibited photostimulation-induced IPSCs. Moreover, the average amplitude of these induced IPSCs in GABAergic neurons (94.1 ± 18.2 pA, mean ± SEM, *n* = 28 neurons) was much larger than that in non-GABAergic neurons (18.9 ± 4.4 pA, mean ± SEM, *n* = 3 neurons). Finally, the short latency of induced-IPSCs in GABAergic neurons (3.2 ± 0.2 ms) indicated that CEA^MOR^ neurons mainly made monosynaptic inputs to PB GABAergic neurons (Fig. 6D). Thus, the major synaptic inputs to the PB from CEA^MOR^ neurons were monosynaptic inhibitory synapses of PB GABAergic neurons.

Tachykinin receptor 1 (*Tacr1*)-expressing neurons in the PB (PB*^Tacr1^* neurons), which are mostly glutamatergic, are known to be critical in relaying nociceptive information associated with nociception from the spinal cord [37]. We hypothesized that the activation of the CEA^MOR^-PB pathway during persistent hyperalgesia could recruit PB*^Tacr1^* neurons. If this were true, CFA induction of persistent hyperalgesia should increase the activity of PB*^Tacr1^* neurons, whereas inhibition of PB*^Tacr1^* neurons would alleviate CFA-induced persistent hyperalgesia. Using fiber photometry to measure the calcium response of PB*^Tacr1^* neurons to paw pinch in *Tacr1*-flpo mice, we found that CFA injection significantly increased pinch-induced responses in ipsilateral but not contralateral PB*^Tacr1^* neurons, as compared with those found in the same group of mice before CFA treatment (Fig. 6E–I). Furthermore, pharmacogenetic suppression of these PB*^Tacr1^* neurons alleviated CFA-induced persistent hyperalgesia (Fig. 6J–M) without affecting locomotor activity (Fig. 6N). Next, we examined the synaptic connectivity between GABAergic neurons in the PB and PB*^Tacr1^* neurons using a *Gad2*-Cre^+^/*Tacr1*-flpo mouse line that could express ChR2 and mCherry in GABAergic and PB*^Tacr1^* neurons, respectively, via local viral injection in the PB (Fig. 6O and P, see Methods). We recorded local photostimulation-induced IPSCs in PB*^Tacr1^* neurons and found that PB GABAergic neurons made monosynaptic inhibitory connections with PB*^Tacr1^* neurons (Fig. 6Q and R). To determine the functional connection between PB neurons receiving CEA projections and PB*^Tacr1^* neurons, we performed a whole-cell patch-clamp recording of PB*^Tacr1^* neurons and found that those PB neurons that received CEA projections made monosynaptic GABAergic synapses with PB*^Tacr1^* neurons (Fig. S9). Thus, CEA^MOR^ neurons could gate the activity of PB*^Tacr1^* neurons by making monosynaptic inhibitory synapses on PB GABAergic neurons that directly innervate PB*^Tacr1^* neurons.

## DISCUSSION

**Figure 7.**
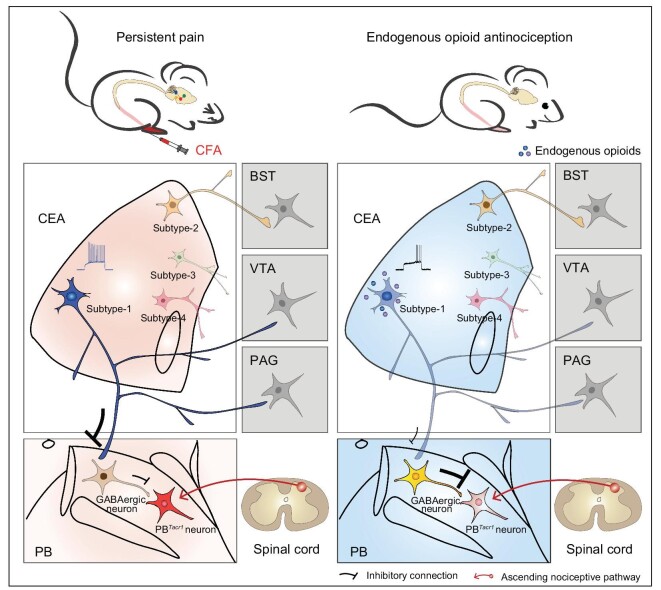
Diagram illustrating the neural circuit underlying endogenous opioid antinociception. The neural activity of CEA^MOR^ neurons was increased after CFA treatment. Endogenous opioids in the CEA suppressed CEA^MOR^ neurons. Subtype-1 CEA^MOR^ neurons gate the activity of PB*^Tacr1^* neurons, which play an important role in relaying nociceptive information, via a di-synaptic disinhibitory pathway, and underlie endogenous opioid antinociception.

In this study, we identified a neural circuit that was essential for mediating endogenous opioid antinociception during CFA-induced persistent hyperalgesia. We found that endogenous opioid antinociception was mediated by MORs expressed in CEA inhibitory projection neurons. Using single-neuron projectome analysis, we defined the projection subtypes of CEA^MOR^ neurons, each of which could project to multiple brain regions. Based on the projectome patterns of single CEA^MOR^ neurons, we showed that PB-projecting axon branches of subtype-1 neurons were involved in endogenous opioid antinociception. Further neural circuit analysis of the CEA-PB pathway showed that a disinhibitory local circuit within the PB involving GABAergic interneurons and *Tacr1*-expressing neurons was responsible for mediating the gating of persistent hyperalgesia by CEA^MOR^ neurons. The use of pharmacogenetic and optogenetic manipulations and single-neuron connectome analysis allowed the elucidation of the CEA-PB circuit mechanism underlying endogenous opioid antinociception. Thus, endogenous opioids could inhibit CEA^MOR^ neurons, which in turn suppress CEA^MOR^-PB disinhibitory circuits, leading to the alleviation of persistent hyperalgesia by recruiting PB*^Tacr1^* neurons (Fig. 7).

### MOR activation in the CEA for endogenous opioid antinociception during persistent hyperalgesia

In the present study, we demonstrated the indispensable role of MORs expressed in the CEA in endogenous opioid antinociception in a mouse model of persistent inflammatory hyperalgesia. This was evidenced by the data showing that selective deletion of MORs in the CEA impaired recovery from CFA-induced persistent hyperalgesia. The important role of MORs expressed in the CEA was further supported by data showing that endogenous opioid antinociception could be largely restored in both male and female MOR-null mice by selectively restoring MOR expression in CEA neurons. Given that similar results were also observed in female mice, the majority of our experiments exclusively utilized male mice, aiming to minimize the use of experimental animals. Our strategy for re-expressing MORs helped to restore the original pattern of MOR expression [6], which is difficult to achieve by transgenic approaches [8,49]. This genetic approach could also discriminate the effects mediated by pre- vs. postsynaptic MORs, which have been difficult to examine by traditional pharmacological approaches. Our results suggest that CEA is important for pain modulation [17,50], and is involved in endogenous opioid analgesia [51,52]. After re-expression of MOR in *Oprm1*^KI/KI^ mice, the recovery of the pain threshold did not return to basal level, indicating that the concurrent involvement of other MOR-expressing neurons is necessary to achieve full pain resolving, although MOR activity in CEA neurons likely plays an essential role.

An important issue of MOR involvement in endogenous opioid antinociception is whether MORs in the CEA are activated by locally released opioids. Enhanced release of opioids in the brain has been observed in several situations, such as stress-induced analgesia, and the effects could be blocked by naloxone [3,5,7]. Consistently, our pharmacological experiments showed that bilateral CEA infusion of naloxone, a competitive opioid antagonist in wild-type mice, blocked endogenous opioid antinociception, indicating the requirement of opioid receptor activation by endogenous opioid peptides during CFA-induced persistent hyperalgesia. Specific involvement of MOR activation was further demonstrated by the effect of the MOR-specific neutral antagonists CTAP and 6β-naltrexol on CEA-Cre/*Oprm1*^KI/KI^ mice, in which MORs were specifically re-expressed in the CEA neurons of MOR null mice. It was also proven that endogenous MOR activity in the CEA is involved in preventing the transition from acute to chronic pain [51]. On the other hand, constitutive activation of MOR in the spinal cord could also be involved in endogenous analgesia [13], in which the effect of presynaptic MORs expressed in descending fibers cannot be ruled out [53,54]. This mechanism might not be involved in CEA, as we found that endogenous opioid antinociception was blocked by neutral MOR antagonists CTAP and 6β-naltrexol, which are known to be ineffective in blocking constitutively active MORs. Thus, our data suggest that locally released opioids in the CEA are required for activating MORs on CEA^MOR^ neurons, leading to endogenous opioid antinociception.

### CEA^MOR^ neurons gate endogenous opioid antinociception

The role of CEA^MOR^ neurons in endogenous opioid antinociception was evidenced by the increased excitability of CEA^MOR^ neurons during CFA-induced persistent hyperalgesia and decreased excitability when the hyperalgesia subsided. The activation of MORs is known to suppress neuronal activity via the G_αi_-mediated intracellular pathway [1], which was confirmed in CEA^MOR^ neurons. Since pharmacogenetic activation of CEA^MOR^ neurons aggravated persistent hyperalgesia in our mouse model, it is likely that increased activity of CEA^MOR^ neurons contributes to persistent hyperalgesia and that locally released opioids alleviate persistent hyperalgesia by suppressing the activity of these neurons. Consistent with this idea, pharmacogenetic suppression of CEA^MOR^ neuron activity mimicked the effect of MOR activation and alleviated persistent hyperalgesia. In Fig. 3A, we used MOR-iCreER^T2-/−^ mice; iCre^ERT2^ was inserted into the exon 2 of *Oprm1* in both alleles, thus there is no MOR expressed. This strategy avoids interference by endogenous MOR activation that could suppress neuronal activity via inhibitory Gαi. Therefore, pharmacogenetic inhibition of CEA^MOR^ neurons even without MOR expression could produce analgesia during inflammatory hyperalgesia, which mimics the antinociceptive effect of endogenous opioids. However, for the pharmacogenetic activation experiments in Fig. 3E, we would like to keep the animal in a status similar to the wild-type mice. So, in Fig. 3E, we used MOR-iCreER^T2+/−^ mice; iCre^ERT2^ was inserted into the exon 2 of *Oprm1* in one allele, thus MOR is still expressed in MOR-expressing neurons. The notion that the CFA-induced hyperalgesia results from an increase of neuronal excitability of CEA^MOR^ neurons, and that endogenous opioids suppress this increase, was further supported by our electrophysiological recording results, which show that naloxone elevated firing of CEA^MOR^ neurons at day 21 after CFA injection when the mice had recovered from persistent hyperalgesia (Fig. 2T–W). These results are consistent with previous findings that implicated the CEA in endogenous analgesia during persistent hyperalgesia [55,56].

Several studies have shown that the right CEA exhibits an overwhelmingly pro-nociceptive function across multiple pain models [50,57–63], thus in most of our experiments, we recorded the neuronal activity of CEA^MOR^ neurons in the right CEA. We found that the response of CEA^MOR^ neurons in the right CEA to right but not left hindpaw pinch was markedly increased after intraplantar CFA injection compared with the responses in the same group of mice before CFA treatment. These results support the notion that the function in the CEA may be lateralized [50,58,60]. However, we have not specifically addressed the issue of lateralization in the current study. Moreover, our results are in line with previous studies showing that the right CEA are critical for nociception [50,57,60,62,64], although these results are inconsistent with the previous reports showing that the activity of right CEA neurons increased in response to noxious stimulus of both left and right hindpaws [58,60]. The possible reason is that our recording site is different from the previous studies. The signal we collected was mainly from the CEAm and CEAl (Fig. S3A and B), but not CEAc, which is the subregion showing increased response to noxious stimulus in previous studies [57,60]. Together, these results indicate that subregions of right CEA could be differentially involved in processing nociceptive signals of ipsilateral and contralateral sides.

### Projectome-guided neural circuit analysis

As the major output nucleus of the amygdala, the CEA has been extensively studied [65]. Most previous studies on the neural mechanisms of the CEA in different physiological processes (i.e. fear, pain modulation) were mostly based on molecularly defined subtypes of CEA neurons [17,66,67]. Using single-neuron projectome analysis, we demonstrated that CEA^MOR^ neurons could be further classified into subtypes based on the distinct patterns of their axon projections. This analysis revealed the structural complexity of molecularly defined neurons, particularly in their branching patterns and preferential projection targets. The information on projectome-based subtypes further guided our dissection of the circuit basis of endogenous opioid antinociception. We found that CEA^MOR^ neurons were involved in endogenous opioid antinociception in a subtype-dependent manner. This was evidenced by the finding that optogenetic suppression of axon projections at the major target of subtype-1 but not subtype-2 CEA^MOR^ neurons could alleviate persistent hyperalgesia, mimicking endogenous opioid antinociception.

Our projectome analysis showed that the axons of CEA^MOR^ neurons exhibited a complex branching pattern targeting multiple brain regions and showed target preferences for each CEA^MOR^ subtype. We demonstrated that subtype-1 CEA^MOR^ neurons were engaged in endogenous opioid antinociception via their axon projections to the PB, which is widely known to be an important central relay for nociceptive information processing [37,68]. This substantiated the notion that the CEA-PB pathway is involved in pain modulation [39,69,70] by identifying the CEA neuron subtype involved in the modulation of endogenous antinociception. Notably, subtype-1 neurons have axon branches that target other brain areas besides PB, such as PAG. Although PAG, as a key component of the descending pain modulatory circuit, has previously been strongly implicated in endogenous analgesia, both GABAergic and glutamatergic neurons in the PAG were found receiving GABAergic inputs from CEA [41,43,44]. Our data showed that optogenetic suppression of the projection from CEA^MOR^ neurons to PAG induced thermal and mechanical hyperalgesia and exhibited the opposite role to endogenous opioid antinociceptive effects, which is in line with a recent study [71]. The other major target, VTA, is not involved in endogenous opioid antinociception either. This projectome-guided circuit analysis underscores the importance of addressing distinct functions performed by axon branches of the same type of neuron projecting to different target regions. Notably, the projectome information on the multiple targets of single axons provides the basis for understanding how a single group of neurons may coordinate the activity and function of multiple neural pathways.

In the current study, we used two approaches to determine the projection pattern of CEA^MOR^ neurons. There appear to be some discrepancies between these two approaches. This is most likely due to the difference in measurement accuracy of the two approaches. Specifically, the dense labeling approach showed that the majority of tdTomato-labeled neurons were located in the right CEA. However, some tdTomato-labeled neurons were also detected in nearby areas (basomedial amygdalar nucleus, BMA) (Fig. S5B), which indicated that the virus unavoidably spread to nearby areas in our experiment. It was hard to distinguish whether these detected tdTomato-labeled synaptic terminals originated from the CEA or nearby areas. Thus, we could not exclude contamination from these neurons in nearby areas. For examination of the projectome of CEA^MOR^ neurons at single-neuron resolution, we used HD-fMOST technology. In this experiment, neurons in nearby areas were excluded from the analysis, thus we could obtain precise projection information of CEA^MOR^ neurons. The resolution in plotting represents another reason for the discrepancy. The projection in the ACB could be an example. In the current study, the synaptic terminals of CEA^MOR^ neurons densely labeled with tdTomato were detected in the ACB, although the projection intensity was relatively weak in single cell projectome analysis (Fig. S5). In single-neuron projectome analysis, only 5 out of 67 neurons sent sparse projection to the ACB. Overall, the projection patterns detected with the two methods were consistent. These projectome to ACB were below the threshold for plotting, therefore they were not included in Fig. 4.

Previous studies combining single-cell transcriptional profiling with axonal reconstruction suggest that there is no one-to-one congruent correspondence between the transcriptome- and projectome-defined subtypes of neurons [32,72,73]. Since the input and output connectivity of neurons is generally more stable than the transcriptomic patterns and is a critical factor in defining a neuron's functional role within the circuit, it is important to map single-neuron projectomes and define projection-based neuronal subtypes as a basis of neural circuit analysis. Large-scale efforts are now devoted to mapping single-cell connectomes in mammalian brains [22–25], although functional analysis based on connectome information has been rare [32]. Our study used projectome analysis to further classify a molecularly defined group of CEA neurons and further delineated their functional distinction. These structurally defined subtypes are likely to express distinct transcriptomes that remain to be determined. Identification of specific and stable molecular markers for projectome-defined neuron subtypes could help to further probe the functional role of each subtype and gain deeper insights into the formation and modulation of specific neural circuits.

It has been shown that after NpHR activation, there could be evoked GABA-mediated excitation [74]. However, in our behavior test, we performed both pharmacogenetic and optogenetic manipulations to determine the functional role of CEA^MOR^-PB projection in endogenous antinociceptive action, and the consistent results were observed, indicating the reliable efficacy of both approaches. Thus, this would not affect our conclusions.

### Limitations

In the current study, due to different purposes, the experiments were performed on different days after CFA injection. CFA-induced hyperalgesia was characterized by a change in nociceptive sensitivity. After CFA injection, mice would exhibit hyperalgesia, followed by slow recovery to nearly basal level 3–4 weeks later. This recovery was shown to be mediated by endogenous activation of MORs [6,13,51]. Thus, most of the behavioral tests examining the effect of different manipulation on recovery of hyperalgesia were performed across the 49 days of the experiment. However, this does not apply to the experiments in Fig. 3A–D and Fig. 5, whose aim was to mimic the MOR-mediated endogenous opioid antinociception by pharmacogenetic or optogenetic suppression of CEA^MOR^ neurons or the CEA-PB circuit. These manipulations could only be performed in the early days but not at a later stage, when the hyperalgesia had already recovered to basal level. For the prolonged pharmacogenetic activation of CEA^MOR^ neurons, CNO was injected every other day, and the behavioral test was terminated at day 21 after CFA injection, when the hyperalgesia largely recovered (Fig. 3H and I). For testing the effect of MOR antagonists in blocking the endogenous opioid activation, we tested the effect of different antagonists at late stages, when the endogenous opioid system was activated (Fig. 1S, V and W). In some experiments, the same batch of mice were used in testing different antagonists or the same manipulation on different noxious stimuli, thus these experiments were performed on different days.

In summary, our findings revealed a specific neural circuit underlying the alleviation of persistent inflammatory hyperalgesia by endogenous opioids, paving the way for target-specific treatments of chronic pain via pharmacological and neuromodulatory approaches. Since the PB is a critical relay nucleus associated with pain processing, the circuit mechanism we identified in this study could be a general self-regulatory mechanism for modulating pain, enabling flexible behavior even under pain conditions. Our study also illustrates a new paradigm for single-neuron projectome-guided analysis of diverse circuit functions served by different axon branches of the same neuronal subtype.

## Supplementary Material

nwae195_Supplemental_Files
