# Supplementary material for: Single-neuron projectome-guided analysis reveals the neural circuit mechanism underlying endogenous opioid antinociception
Source: Natl Sci Rev. 2024 Jun 4;11(7):nwae195. doi: 10.1093/nsr/nwae195 (PMC11264302; doi:10.1093/nsr/nwae195)
Supplement: nwae195_Supplemental_Files [file nwae195_supplemental_files.zip › SI-R1.docx]

**METHODS**

**CONTACT FOR REAGENT AND RESOURCE SHARING**

Further information and requests for sources should be directed to the Lead Contact, Yan-Gang Sun (yangang.sun@ion.ac.cn).

**Animals**

Male C57BL/6J, *Oprm1*^fl/fl^, *Oprm1*^KI/KI^, MOR-iCreER^T2^ (Zhang et al., 2020), Ai9 (*Rosa26^tdTomato^*, JAX007909), *Gad2*-T2a-NLS-mCherry (JAX023140), *Gad2*-Cre (JAX010802) and *Tacr1*-flpo (Deng et al., 2020) and female *Oprm1*^KI/KI^ mice at the age of 8-10 week-old were used for experiments. MOR-iCreER^T2^/*Gad2*-T2a-NLS-mCherry mice were generated by crossing MOR-iCreER^T2^ mice with *Gad2*-T2a-NLS-mCherry mice. All mice were raised on a 12-h light/ dark cycle (lights on at 7:00 am) with *ad libitum* food and water. All procedures were approved by the Animal Care and Use Committee of the Center for Excellence in Brain Science & Intelligence Technology, Chinese Academy of Sciences, China.

**METHOD DETAILS**

**Stereotaxic injection**

Mice were anesthetized by intraperitoneal injection of anesthetic mixture of Zoletil (Tiletamine hydrochloride: 15 mg/kg; Zalazepam hydrochloride: 15 mg/kg; purchased from Virbac S.A) and Xylazine hydrochloride (80 mg/kg, purchased from Huamu Veterinary Medicine Co., Ltd.). All the stereotaxic injection was performed with a stereotaxic apparatus, and oculentum was applied to maintain eye lubrication. Virus was injected at a rate of 10-50 nl/min using an air pressure system by connecting to borosilicate glass pipettes (tip diameter of 10-30 μm) or oil pressure system by connecting to glass pipettes (tip diameter of 10-20 μm) filled the mineral oil. After the injection, the pipette was left in position for another 5-10 min before withdrawal. Mice were allowed to recover from anesthesia on a heating blanket before returning them to their home cages. To relieve postoperative pain and prevent infection, Tolfenamic acid (0.05%, 0.1 ml per mice) and Ceftriaxone sodium (100 mg/kg) were intraperitoneally injected continuously for three days after surgery.

**Surgery for the viral injection and optical fiber or cannula implantation into the brain**

Injection sites for the CEA, ACB and PB were bregma: -1.46 mm, lateral: ±2.75 mm, depth: -4.52 mm; bregma: 0.98 mm, lateral: ±1.20 mm, depth: -4.60 mm; bregma: -5.40 mm, lateral: ±1.25 mm, depth: -3.33 mm, respectively. All viruses were injected with a volume of 200-400 nl/site.

To determine the functional role of MORs expressed in the CEA in hyperalgesia, AAV2/8-hSyn-Cre-EGFP (titer: 5.90 × 10^12^ v.g./ml, Taitool) or AAV2/9-hSyn-EGFP (titer: 2.73 × 10^12^ v.g./ml, Taitool) virus was bilaterally injected into the CEA of *Oprm1*^fl/fl^ or *Oprm1*^KI/KI^ mice. To test the effect of opioid receptor antagonists, cannula was implanted above bilateral CEA and the opioid antagonists were infused locally via an injection tube.

To examine the neuronal activity of CEA^MOR^ neurons during noxious stimuli, AAV2/9-hSyn-DIO-GCaMP6s-WPRE-pA (titer: 4.27 × 10^12^ v.g./ml, Taitool) virus was injected into the right CEA of MOR-iCreER^T2+/-^ mice. Optical fiber was implanted 0.05-0.10 mm above the injection site. To determine the effect of pharmacogenetic activation of CEA^MOR^ neurons on the response of these neurons to pinch stimuli, AAV2/9-hSyn-DIO-GCaMP6s-WPRE-pA (titer: 4.5 × 10^12^ v.g./ml, Taitool) and AAV2/9-hSyn-DIO-hM3Dq-mCherry (titer: 4.42 × 10^12^ v.g./ml, Taitool) was injected in the right CEA of MOR-iCreER^T2+/-^ mice.

To determine whether the phenomenon of no longer increasing in neuronal excitability of CEA^MOR^ neuron was due to the suppression of the CEA^MOR^ neuron by the released endogenous opioids, AAV2/9-Ef1α-DIO-EYFP (titer: 3.83 × 10^12^ v.g./ml, Taitool) virus was injected into the right CEA of MOR-iCreER^T2+/-^ mice. Tamoxifen induction was performed every other day for 3 days. Two weeks after viral injection, CFA was injected into the right hind paw of these mice. After 21 days, the whole-cell patch-clamp was performed on EYFP-labeled CEA^MOR^ neurons and the intrinsic properties of “late-firing” CEA^MOR^ neurons were recorded 30 min after naloxone (3 mg/kg, intraperitoneal, i.p.) or saline injection.

To determine the effect of activation of MOR by DAMGO on the excitability of CEA^MOR^ neurons, AAV2/9-Ef1α-DIO-EYFP (titer: 3.83 × 10^12^ v.g./ml, Taitool) virus was injected into the right CEA of MOR-iCreER^T2+/-^ mice. Tamoxifen induction was performed every other day for 3 days. Two weeks after viral injection, the whole-cell patch-clamp was performed on EYFP-labeled CEA^MOR^ neurons and the intrinsic properties of CEA^MOR^ neurons were recorded before and after DAMGO (1 μM, Tocris, 1171) application.

To manipulate the neuronal activity of CEA^MOR^ neurons with designer receptors exclusively activated by designer drugs (DREADDs), AAV2/5-hSyn-DIO-hM4Di-mCherry (titer: 5.40 × 10^12^ v.g./ml, Addgene) or AAV2/9-Ef1α-DIO-EYFP (titer: 3.83 × 10^12^ v.g./ml, Taitool) virus was bilaterally injected into the CEA of MOR-iCreER^T2-/-^ mice. AAV2/9-hSyn-DIO-hM3Dq-mCherry (titer: 4.33 × 10^12^ v.g./ml, Taitool) or AAV2/9-Ef1α-DIO-EYFP (titer: 3.83 × 10^12^ v.g./ml, Taitool) virus was injected into the right CEA of MOR-iCreER^T2+/-^ mice.

To ablate CEA^MOR^ neurons, AAV2/9-CAG-DIO-taCasp3-TEVp (titer: 4.95 × 10^12^ v.g./ml, Taitool) or AAV2/9-Ef1α-DIO-EYFP (titer: 3.83 × 10^12^ v.g./ml, Taitool) virus was bilaterally injected into the CEA of MOR-iCreER^T2^ × Ai9 mice. To ablate ACB^MOR^ neurons, AAV2/9-CAG-DIO-taCasp3-TEVp (titer: 4.20 × 10^12^ v.g./ml, Taitool) or AAV2/9-Ef1α-DIO-EYFP (titer: 3.83 × 10^12^ v.g./ml, Taitool) virus was bilaterally injected into the ACB of MOR-iCreER^T2^ × Ai9 mice.

To examine the output of CEA^MOR^ neurons, AAV2/8-hSyn-DIO-Synaptophysin-tdTomato (titer: 1.71 × 10^13^ v.g./ml, Taitool) was injected into the right CEA of MOR-iCreER^T2+/-^ mice.

To trace the projection pattern of CEA^MOR^ neurons by fluorescence micro-optical sectioning tomography (fMOST) scanning, the sparse labeling virus (AAV2/9, titer: 1.41 × 10^13^ v.g./ml, BC-SL003, Brain Case Biotech Co., Ltd), produced by co-packaging the DNA cassettes of AAV-Ef1α-DIO-flpo and AAV-Ef1α-fDIO-EYFP at a ratio of 1:8000 (Ref (Sun et al., 2020)), was injected into the right CEA of MOR-iCreER^T2+/-^ mice.

To inhibit the projection from CEA^MOR^ neurons to PB, AAV2/5-hSyn-DIO-hM4Di-mCherry (titer: 5.40 × 10^12^ v.g./ml, Addgene) was injected into the right CEA of MOR-iCreER^T2-/-^ mice and cannula was implanted above the right PB, AAV2/9-Ef1α-DIO-EYFP (titer: 3.83 × 10^12^ v.g./ml, Taitool) was used as a control. We also applied optogenetics to inhibit the pathway, AAV2/8-Ef1α-DIO-eNpHR3.0-EYFP (titer: 6.10 × 10^12^ v.g./ml, Taitool) or AAV2/9-Ef1α-DIO-EYFP (titer: 3.83 × 10^12^ v.g./ml, Taitool) was bilaterally injected into the CEA of MOR-iCreER^T2+/-^ mice and optical fibers were implanted above the bilateral PB.

For optogenetic suppression the projection from CEA^MOR^ neurons to PAG, VTA or BST, AAV2/8-Ef1α-DIO-eNpHR3.0-EYFP (titer: 6.10 × 10^12^ v.g./ml, Taitool) or AAV2/9-Ef1α-DIO-EYFP (titer: 3.83 × 10^12^ v.g./ml, Taitool) was injected into the right CEA of MOR-iCreER^T2+/-^ mice and optical fiber was implanted above the right PAG (bregma: -4.75 mm, lateral: -0.55 mm, depth: -2.20 mm), VTA (bregma: -3.3 mm, lateral: -0.5 mm, depth: -4.52 mm) or BST (bregma: 0.2 mm, lateral: -0.75 mm, depth: -4.52 mm).

To examine the neuronal activity of *Tacr1^+^* neurons in PB (PB*^Tacr1^* neurons) during noxious stimuli before and after CFA application, AAV2/9-Ef1α-fDIO-GCaMP6s-WPRE-pA (titer: 8.4 × 10^12^ v.g./ml, Taitool) was injected into the right PB of *Tacr1*-flpo mice (in this experiment, a MOR-iCreER^T2^ allele is also present in this mouse line) and the optical fiber terminal was implanted 0.05-0.10 mm upper to the injection site.

To determine the functional role of PB*^Tacr1^* neurons in CFA-induced persistent hyperalgesia, AAV2/9-Ef1α-fDIO-hM4Di-mCherry (titer: 4.28 × 10^12^ v.g./ml, Taitool) or AAV2/9-Ef1α-fDIO-mCherry (titer: 3.82 × 10^12^ v.g./ml, Taitool) virus was bilaterally injected into the PB of *Tacr1*-flpo mice.

To define the synaptic connections between CEA^MOR^ neurons and PB neurons, AAV2/9-Ef1α-DIO-hChR2(H134R)-EYFP (titer: 3.06 × 10^12^ v.g./ml, Taitool) was injected into the right CEA of MOR-iCreER^T2+/-^ and MOR-iCreER^T2^/*Gad2*-T2a-NLS-mCherry mice.

To examine the functional synaptic connection between CEA-PB projecting neurons and PB*^Tacr1^* neurons, AAV2/1-CMV-bGI-Cre-EGFP-pA (titer: 1.53 × 10^13^ v.g./ml, Taitool) was injected into the right CEA and AAV2/9-Ef1α-fDIO-mCherry (titer: 3.80 × 10^12^ v.g./ml, Taitool) was injected into the right PB of *Tacr1*-flpo mice. One week later, AAV2/9-Ef1α-DIO-ChR2-EYFP (titer: 3.54 × 10^12^ v.g./ml, Taitool) was injected in the right PB of *Tacr1*-flpo mice.

To examine the functional synaptic connection between GABAergic neurons and *Tacr1^+^* neurons in PB, mixed viruses of AAV2/9-Ef1α-DIO-hChR2(H134R)-EYFP (titer: 1.53 × 10^12^ v.g./ml, Taitool) and AAV2/9-Ef1α-fDIO-mCherry (titer: 2.00 × 10^12^ v.g./ml, Taitool) were injected into the right PB of *Gad2*-Cre/*Tacr1*-flpo mice.

To determine the relationship between PB-projecting CEA^MOR^ neurons and PKCδ^+^ neurons in the CEA, AAV2/2-Retro-CAG-Flex-Flpo (titer: 7.86 × 10^12^ v.g./ml, Taitool) and AAV2/9-Ef1α-fDIO-EYFP (titer: 2.20 × 10^12^ v.g./ml, Taitool) was injected into the right PB and CEA, respectively, of MOR-iCreER^T2+/-^ mice.

**Tamoxifen induction**

To induce the expression of Cre recombinase in MOR-iCreER^T2^ × Ai9, MOR-iCreER^T2+/-^ and MOR-iCreER^T2-/-^ mice, tamoxifen (Sigma, T5648) was dissolved in sunflower oil at room temperature and was injected intraperitoneally from 3-5 days after virus injection for 3 times every other day (225 mg/kg). All the experiments were performed 4-6 weeks after tamoxifen induction unless otherwise noted.

**Complete Freund's adjuvant (CFA)-induced persistent hyperalgesia**

Mice received subcutaneous injection of CFA (50%, 20 μl, Sigma, F5881) into the plantar of the right hind paws. During persistent hyperalgesia, we tested the thermal and mechanical hyperalgesia on the ipsilateral paws by Hargreaves test and von Frey test, respectively (Zhang et al., 2020). For testing thermal sensitivity, we applied Hargreaves apparatus (Ugo Basile, Comerio, Italy) to measure the paw withdrawal latency with the cut-off time of 20 s to prevent tissue injury. To determine the mechanical hyperalgesia, we used a series of von Frey filaments to determine the 50% hind paw withdrawal threshold by the modified Dixon’s up-down method (Chaplan et al., 1994). The nociceptive threshold was calculated from average of two trials separated by 30-min interval.

To determine whether MOR activation in CEA neurons during persistent hyperalgesia is mediated by opioids released endogenously in CEA or by an agonist-independent constitutive MOR activation, MOR-specific neutral antagonists (CTAP, 300 ng/0.5 μl; 6β-naltrexol, 1 μg/0.5 μl), or a competitive opioid receptor antagonist naloxone (200 ng/0.5 μl) was locally infused into bilateral CEA on day 42 or day 49 after CFA application in wild-type mice or *Oprm1*^KI/KI^ mice injected with AAV2/8-hSyn-Cre-EGFP into the bilateral CEA. The mechanical sensitivity was tested on the ipsilateral hind paws before and 15, 30, 60, 120, 180, 240 min after drug infusion.

**Nociceptive behavior tests**

The nociceptive behavioral tests were performed as described previously (Zhang et al., 2020). The tail immersion test was performed by gently restraining mice in a cotton towel and dipping one third of the tail from tip into a water bath of 48 °C. We recorded the tail flick latency and a maximal cut-off time of 20 s was set to avoid tissue damage.

For the hot plate test, mice were placed on the hot plate (Ugo Basile, Comerio, Italy) at 52 °C, and the first appearance of hind paw lifting, jumping or licking was recorded with a cut-off time of 45 s to avoid tissue damage.

Randall-Selitto Analgesy-meter (Ugo Basile, Varese, Italy) was used to measure tail flick threshold to noxious mechanical stimulation. Mouse was gently held and a mechanical force that increases linearly with time was applied directly to the dorsal surface of the one third of the tail from tip. A maximal cut-off force of 250 g was set to avoid tissue damage. And then we counted the tail flick threshold as the force at which the mouse flicked its tail.

For the von Frey test, we perpendicularly stimulated mouse hind paw by a series of von Frey hairs with logarithmically incrementing stiffness (0.04-1.4 grams, Stoelting, Wood Dale, IL). Every filament was applied 5 times in one trial with intervals more than 5 s. And the paw withdrawal times were recorded to calculate paw withdrawal frequency which presented the mechanical threshold.

**Open field test**

Open field test was performed to evaluate the locomotor activity (Zhang et al., 2020). Mice were acclimated for at least 30 min in the testing room. After habituation, mice were placed in the center of the open field chambers (40 × 40 × 40 cm) and videotaped individually for 10 min. Average speed, total travel distance and track visualization was analyzed by EthoVision XT 11.5 (Noldus, Wageningen, Netherlands) software in a 10-min period.

**Rotarod test**

Rotarod test was performed to evaluate the motor ability (Mu et al., 2017). On the first two days, mice were placed on a rotarod apparatus that accelerates 5-20 revolution per minute (r.p.m) for 5 min to train these mice maintaining their balancing walking. On the third day, rod accelerated 5-40 r.p.m., and mice were tested twice with a maximum time of 300 s. The latency of mouse to fall off was recorded.

**Pharmacogenetic manipulations**

For *in vivo* pharmacogenetic inhibition of CEA^MOR^ neurons on nociceptive sensitivity, MOR-iCreER^T2-/-^ mice were bilaterally injected with AAV2/5-hSyn-DIO-hM4Di-mCherry or AAV2/9-Ef1α-DIO-EYFP virus into the CEA. CNO (3 mg/kg, Sigma) was intraperitoneally injected every other day. Behavioral tests were performed before and 30 min after CNO application on the specific days in the graphical representation of data.

For *in vivo* pharmacogenetic activation of CEA^MOR^ neurons on nociceptive sensitivity, MOR-iCreER^T2+/-^ mice were injected with AAV2/9-hSyn-DIO-hM3Dq-mCherry or AAV2/9-Ef1α-DIO-EYFP virus into the right CEA. For nociceptive tests, CNO (1 mg/kg, Sigma) was intraperitoneally injected 30 min before the tests, and for the CFA-induced persistent hyperalgesia, CNO (1 mg/kg, Sigma) was intraperitoneally injected every other day from 2 h after CFA application.

For *in vivo* pharmacogenetic suppression of the projection from CEA^MOR^ neurons to PB on CFA-induced persistent hyperalgesia, MOR-iCreER^T2-/-^ mice were injected with AAV2/5-hSyn-DIO-hM4Di-mCherry or AAV2/9-Ef1α-DIO-EYFP virus into the right CEA and were implanted cannula above the right PB. CNO (0.5 μg/ 0.5 μl, Sigma) was injected into the PB and nociceptive behavioral tests were performed 30 min after CNO application.

For *in vivo* pharmacogenetic inhibition of PB*^Tacr1^* neurons in CFA-induced persistent hyperalgesia, *Tacr1*-flpo mice were bilaterally injected with AAV2/9-Ef1α-fDIO-hM4Di-mCherry or AAV2/9-Ef1α-fDIO-mCherry virus in the PB. After virus expression for 4 weeks, CFA-induced persistent hyperalgesia model was developed and nociceptive behaviors were performed before and on specific days after CFA application. CNO (3 mg/kg, Sigma) was intraperitoneally injected every other day. Behavioral tests were performed before and 30 min after CNO injection.

**Optogenetic manipulations**

For *in vivo* optogenetic suppression of the projection from CEA^MOR^ neurons to PB on CFA-induced persistent hyperalgesia, MOR-iCreER^T2+/-^ mice were implanted with optical fibers (NA 0.37) targeting at the bilateral PB (bregma: -5.40 mm, lateral: ± 2.00 mm, depth: -2.84 mm at an angle of 15 degrees) after the bilaterally injection of AAV2/8-Ef1α-DIO-eNpHR3.0-EYFP or AAV2/9-Ef1α-DIO-EYFP virus into the CEA. To evaluate the locomotor activity in open field test for *in vivo* optogenetic suppression of the projection from CEA^MOR^ neurons to PB, PAG, VTA or BST, freely moving mice were videotaped individually for 3 min (light OFF) and then the continuous laser (593 nm, ~10 mW) was delivered for 3 min (light ON), the light was subsequently shut-off for another 3 min (light OFF). To determine the functional role of the projection from CEA^MOR^ neurons to PB on basal nociceptive sensitivity and CFA-induced persistent hyperalgesia, before and on specific days after CFA injection, Hargreaves and von Frey tests were performed on these mice twice with 10-min interval before light stimulation, with continuous laser (593 nm, ~10 mW) stimulation, and after light stimulation, respectively. The mean value of nociceptive threshold was calculated. For *in vivo* optogenetic suppression of the ipsilateral projection from CEA^MOR^ neurons to PAG, VTA or BST on CFA-induced persistent hyperalgesia, MOR-iCreER^T2+/-^ mice were implanted with optical fiber (NA 0.37) targeting at the right PAG (bregma: -4.75 mm, lateral: -0.55 mm, depth: -2.20 mm), VTA (bregma: -3.3 mm, lateral: -0.5 mm, depth: -4.52 mm) or BST (bregma: 0.2 mm, lateral: -0.75 mm, depth: -4.52 mm) after the injection of AAV2/8-Ef1α-DIO-eNpHR3.0-EYFP or AAV2/9-Ef1α-DIO-EYFP virus into the right CEA. Before and on specific days after CFA injection, Hargreaves and von Frey tests were performed on these mice with continuous laser (593 nm, ~10 mW) stimulation.

**Fiber photometry recording**

Mice were anesthetized by injecting anesthetic mixture of Zoletil (Tiletamine hydrochloride: 8.33 mg/kg; Zalazepam hydrochloride: 8.33 mg/kg; purchased from Virbac S.A) and Xylazine hydrochloride (0.05 mg/kg, purchased from Huamu Veterinary Medicine Co., Ltd.).

To record the neural activity of CEA^MOR^ or PB*^Tacr1^* neurons in response to paw pinch stimuli, mice received pinches (5 s) using the clip on the ipsilateral and contralateral hind paws for 3-5 times with an interval of 2 min. Calcium signal was recorded before and 1 day after CFA injection. To determine the effect of pharmacogenetic activation of CEA^MOR^ neurons on the response of these neurons to pinch stimuli, the calcium signal was recorded 4 days before and 30 min after CNO (1 mg/kg, i.p.) injection. The start and stop time of each stimulation was tagged and synchronously sent to the fiber photometry system by triggering TTL (Isolated Pulse Stimulator Model 2100, A-M SYSTEMS). And calcium signal was recorded simultaneously at 1000 Hz using F-scope-G-2 (Biolink Optics). The excitation laser power was set at 14 mW. Data were analyzed and transformed into mat file in MATLAB.

**Data analysis for fiber photometry**

The onset and offset of event stimulation was identified first. Fluorescence values were low-pass filtered at 2 Hz by using a 4^th^ order Butterworth filter with zero-phase distortion and then subtracted the noise signal of the recording system. The resulting values were aligned to event onset, and corresponding fluorescence values in each stimulation trial was derived. The dynamics of fluorescence in each stimulation trial was calculated by

∆F/F = (F-F_0_)/F_0_

F indicates fluorescence values in each time point and F_0_ stands for the median of fluorescence values during baseline window (-5 to 0 s before stimulation). The averaged fluorescence change was visualized by heat plot using jet colormap in MATLAB. To quantify the change of fluorescence values across stimulation period, the event windows were defined (0 to 5 s after the event onset). The averaged fluorescence changes in event window and each baseline were calculated and compared.

**Tissue preparation for histology**

Mice were intraperitoneally injected with anesthetic (Tribromoethanol, 400 mg/kg, Sigma) and perfused transcardially with saline followed by 4% paraformaldehyde (PFA, Sigma). The brains were post-fixed overnight at 4 ˚C in 4% PFA, followed by dehydration in 30% sucrose dissolved in PBS at 4 ˚C for at least 48 h. Sections (20 or 40 μm) were prepared with a cryostat (Leica CM 1950) for further *in situ* hybridization and immunostaining.

**Immunofluorescent staining**

Immunofluorescent staining was performed according to the method described previously (Zhang et al., 2020). Briefly, brain sections were blocked for 1 h at room temperature in PBST (0.3% Triton X-100) with 5% normal donkey serum, followed by primary antibody incubation at 4˚C overnight and secondary antibody at room temperature for 2 h in PBST (0.3% Triton X-100) with 1% normal donkey serum. Primary antibodies used in immunohistochemistry (IHC) were anti-MOR (rabbit, 1:500, Abcam, ab134054), anti-DsRed (rabbit, 1:500, Clontech, 632496), and anti-GFP (rabbit, 1:500, Invitrogen, A11122). Secondary antibodies were donkey anti-rabbit IgG-Cy3 (1:200, Jackson ImmunoResearch Laboratories), and donkey anti-rabbit IgG-Alexa Fluor 488 (1:500; Jackson ImmunoResearch Laboratories).

***In situ* hybridization**

We performed multiple *in situ* hybridization experiments using RNAscope Fluorescent Multiplex Assay with *Oprm1*, *S1c32a1* (Vgat) *Sst,* *Pdyn* and *Prkcd p*robes (Advanced Cell Diagnostics, 544731-C2, 319191-C1, 404631-C3, 318771-C1, 441791-C2) to determine the identity of CEA^MOR^ neurons as described previously (Zhang et al., 2020). Briefly, tissue sections were mounted on slides and heated for 2 h at 60 °C and kept at -80 °C before experiments. At room temperature, slides were pretreated with hydrogen peroxide for 20 min and washed in DEPC-H_2_O for 1 min according to manufacturer’s protocol. Then slides were treated by boiling retrieval regent for 7 min, and rinsed in DEPC-H_2_O. Protease digestion was performed in 40 °C HybEZ oven for 15 min. After rinsed in DEPC-H_2_O and washed in DEPC-PBS for 3 min, tissue sections were treated with ethanol for 3 min twice, followed by air-dry process at room temperature. Mixed pre-warmed probes and slides were hybridized in 40 °C HybEZ oven for 2 h. TSA-based signal amplification fluorescent labels were employed.

**Image acquisition**

Images were obtained by using Olympus VS120 microscope, Olympus FV3000 confocal fluorescence microscope and Nikon Tie-A1 plus confocal fluorescence microscope. Confocal images photographed by Olympus FV3000 were cropped in 400 μm × 400 μm areas for co-localization analysis. Cell counting was blindly calculated manually using Fiji (NIH) and Qupath software (version 0.3.2).

**Quantification of axonal projection**

To quantify the axonal projection of CEA^MOR^ neurons, we imaged one section of every three coronal serial sections of 40 μm thickness using microscope (10 × objective lens, Olympus VS120).

For quantification and analysis of whole-brain axonal projections from CEA^MOR^ neurons labeled by AAV2/8-hSyn-DIO-Synaptophysin-tdTomato, we firstly subtracted the background and then normalized all sections of one mouse by multiplying a constant value, which was set to threshold. Pixel above this gray-scale value was recognized as positive signal. Then we measured axon density and projection intensity using ImageJ. The axon density, defined as the area that contains positive tdTomato signal divided by the total area of the target nuclei. The projection intensity, defined as total pixels of positive signal divided by total area for each target nuclei, and then normalized against the intensity of injection site. Raw data from 3 mice were analyzed with Microsoft Excel, and the R Programming Language. Projection pattern was visualized using R packages pheatmap (Version 1.0.12). Downstream brain regions of interest from 3 mice were selected and aligned together with ImageJ, with sections set to 8 bits (value of projection density and intensity level ranging from 0-255). Several representative nuclei were plotted according the logarithm of normalized projection intensity with Python and Microsoft PowerPoint (level of normalized projection intensity ranging from 0 to 5).

**Fluorescence micro-optical sectioning tomography imaging**

Whole-brain mapping of MOR^+^ neurons were achieved by dual-channel fluorescence micro-optical sectioning tomography (dfMOST) imaging (Gong et al., 2016). The tissue preparation was performed as described previously. Briefly, mice were anesthetized and were intracardially perfused with saline followed by 4% PFA (Sigma). The entire brain was post-fixed in 4% PFA at 4 °C for 24 h. And then, each brain was rinsed overnight in a 0.01 M PBS solution that contained 2.5% sucrose at 4 °C and was subsequently dehydrated via a graded series of ethanol mixtures. After dehydration, brains were impregnated with Glycol Methacrylate (GMA, Ted Pella Inc., Redding, CA) by bathing in 70%, 85%, and 100% solutions of GMA water-soluble resin sequentially for 2 h (Ted Pella Inc., Redding, CA) followed by bathing overnight in a 100% GMA solution and an immersion in pre-polymerized GMA at 4 °C for 3 days. Finally, each brain was embedded in a gelatin capsule filled with pre-polymerized GMA and polymerized for 60 h at 60 °C. For whole-brain imaging, the embedded brain samples were imaged by dfMOST system at a voxel size of 0.32 μm × 0.32 μm × 2.0 μm in 3 days for a single mouse brain. Tiles were stitched one by one to get a complete coronal slice, and slices for a whole dataset were obtained from anterior to posterior direction.

**Whole-brain MOR^+^ neurons quantification**

The location of tdTomato-labeled MOR^+^ neurons in the whole brain of MOR-iCreERT2 × Ai9 mouse was defined as follows. After dfMOST imaging, preprocessing including removing stitching stripes and matting the redundant parts out of the brain in each image slice was performed on workstations. Then the whole-brain datasets were resampled at a voxel resolution of 1 μm × 1 μm × 2 μm, and the centers of MOR^+^ neurons in each whole brain were automatically recognized using NeuroGPS software (Quan et al., 2013), saved as three-dimensional coordinates in SWC format. To evaluate the accuracy of automatic locating and counting, we compared the results of automatic recognition with manual positioning. Back-to-back manual validation realized in Amira (v6.1.1, FEI) by three skilled persons was considered as the ground truth. After that, the location results were registered to the Allen Mouse Brain Common Coordinate Framework (CCFv3) (Wang et al., 2020), based on the methodology introduced recently (Ni et al., 2020). The neuron distribution features were quantified using cell densities and nuclei distances in 140 discrete brain regions across the entire extent of the whole brain. Here, cell densities were defined as the number of neurons divided by the voxel volume within each region, and nuclei distances represented the mean distance between each neuron and its closest ones in that region. The visualization of statistical results was done in Amira to get a holistic view of the distribution of MOR^+^ neurons. The whole pipeline of quantification procedure was published previously (Zhang et al., 2017).

**Sample preparation, imaging and tracing for mapping single CEA^MOR^ neuron projectome**

Images for single cell projectome from CEA^MOR^ neurons were performed by high-definition-fMOST (HD-fMOST) system (Zhong et al., 2021). Briefly, the samples were rinsed in 0.01 M PBS three times (12 h each). Then, the samples were dehydrated in a graded series of ethanol (with distilled water): 25%, 50%, 75%, 95%, 100%, 100%, and 100% (2 h each). After dehydration, samples were infiltrated with a graded series of xylene (with pure ethanol): 50% and 100% (2 h each). After infiltration by xylene, samples were infiltrated in a graded series of Lowicryl HM20 (Electron Microscopy Sciences) resin (with xylene): 50%, 75%, 100%, 100%, and 100% (2 h each for the first three and 12 h each for the last two). Samples were transferred into gelatin capsules and then polymerized in a sealed vacuum drying oven by gradient heating (37 °C for 6 h, 42 °C for 8 h, 45 °C for 8 h and 50 °C for 4 h). After infiltration by xylene, samples were infiltrated in a graded series of Lowicryl HM20 (Electron Microscopy Sciences) resin (with xylene): 50%, 75%, 100%, 100%, and 100% (2 h each for the first three and 12 h each for the last two). Samples were transferred into gelatin capsules and then polymerized in a sealed vacuum drying oven by gradient heating (37 °C for 6 h, 42 °C for 8 h, 45 °C for 8 h and 50 °C for 4 h). After HD-fMOST imaging, we obtained two channels of data, one channel is for the fluorescence signal of EYFP (Channel 1, voxel resolution of 0.325 μm × 0.325 μm × 1 μm) and the other channel is for the PI staining (Channel 2, downsampled voxel resolution of 10 μm × 10 μm × 10 μm). The data of channel 1 was turned into three-dimensional visual data after image stitching, and single neuron projectome tracing was performed using FNT software (Gao et al., 2022). Each neuron was manually traced by two independent persons to obtain a comprehensive result. The third person conducts quality inspection on the result to ensure that the tracing accuracy rate is above 90%. After traced the projection pattern of sparely labeled CEA^MOR^ neurons in the whole brain manually, downstream regions labeled with EYFP^+^ fibers were detected. The data of channel 2 is used for registration. The registration process includes linear registration, automatic brain segmentation, manual repair, and nonlinear registration, and the sample is registered to the CCFv3 (Wang et al., 2020). The brain areas involved in automatic brain segmentation are hippocampus, isocortex, thalamus, hypothalamus and caudoputamen. After automatic brain segmentation, these brain areas were manually repaired using ‘3D Slicer’ software. Finally, the tracing results of channel 1 and the registration results of channel 2 are transformed and uploaded to the self-developed software ‘NeuronView’ for neuron search, viewing and subsequent analysis. Clustering analysis was further conducted based on the projection patterns. To construct the connection matrix of all labeled single CEA^MOR^ neuron, we quantified the number of terminals of each neuron in each brain region with data obtained from HD-fMOST tracing. Then we obtained a normalized connection matrix, with connection weight defined as the fraction of total number of terminals, i.e., the number of terminals segmented per each brain region divided by the total number of terminals across all regions. To compare the various projection pattern of these neurons, we performed clustering using NBLAST with Euclidean distance metric and Ward linkages.

**Acute slice preparation**

Brain slice electrophysiology recording was performed as described previously with some modifications (Zhou et al., 2017). Briefly, mice were anesthetized with isoflurane and after decapitation, the brain was isolated in the ice-cold oxygenated cutting solution containing the following (in mM): NMDG 93, HCl 93, NaHCO_3_ 30, HEPES 20, Myo-inositol 3, Sodium-L-ascorbate 1, NaH_2_PO_4_ 1.2, KCl 2.5, MgCl_2_ 10, CaCl_2_ 0.5, Ethyl pyruvate 5, Glucose 25 (pH = 7.3-7.4, 305-310 mOsm). Transverse brain slices containing CEA or PB (250-300 μm) were sectioned in ice-cold oxygenated cutting solution with a vibratome at speed of 0.08 mm/s and vibration amplitude of 1.00 mm. Slices were incubated in the oxygenated recovery solution containing the following (in mM): NaCl 120, NaHCO_3_ 26, Myo-inositol 3, Sodium-L-ascorbate 1, KCl 5, NaH_2_PO_4_ 1.25, MgCl_2_ 1, CaCl_2_ 1, Ethyl pyruvate 5, Glucose 12.5 (pH = 7.3-7.4, 305-310 mOsm) at 32-34 °C for 15 min and then maintained at room temperature for 1 h before electrophysiological recording.

**Slice electrophysiology**

After recovery, the brain slices were placed on poly-L-lysine - coated coverslips and transferred to a recording chamber perfused with oxygenated artificial cerebrospinal fluid (ACSF) at a speed of 4 ml/min. Whole-cell patch-clamp recordings were performed at near-physiological temperatures (32-34 °C). The glass pipette with a resistance of 3-6 MΩ was filled with internal solution containing 0.2% biocytin. Action potentials were generated by current injection from a holding potential of -75 mV. For current-clamp recording in slices, the intracellular solution contained (in mM): K-gluconate 130, KCl 1, MgCl_2_ 1, CaCl_2_ 1, EGTA 11, HEPES 10, Mg-ATP 2, and Na-GTP 0.3 (pH = 7.3-7.4, ~295 mOsm). The rheobase that is defined as the minimal current to evoke an action potential was first determined by depolarization pulses (1 s duration) ranging from 0 to 500 pA in 50 or 100 pA increments on each neuron. The spike frequency was measured by counting the number of spikes within a depolarizing pulse (1 s duration). The first spike-latency was measured as the time between the stimulus onset and the first spike of the response (Dou et al., 2018; Hu et al., 2006). To determine the effect of activation of MOR on the excitability of CEA^MOR^ neurons, the current-clamp recording was performed on EYFP-labeled CEA^MOR^ neurons. And the intrinsic properties of CEA^MOR^ neurons were recorded before and after DAMGO (1 μM, Tocris, 1171) application.

To determine the functional synaptic connection between CEA neurons with PB neurons. Blue light-emitting diode (LED) (475 nm; 10 mW/mm^2^; UHP-Mic-LED-475; Prizmatix, Israel) was used to activate ChR2^+^ fibers (Zhou et al., 2017). The Cs^+^ based internal solution contained (in mM): CsMeSO_3_ 130, HEPES 10, QX-314 2, MgCl_2_ 1, CaCl_2_ 1, EGTA 11, Mg-ATP 2, Na-GTP 0.3 (pH=7.3-7.4, ~295 mOsm) with a holding voltage of -70 or 0 mV, to record the evoked EPSC and IPSC, respectively. To compare the connection between CEA^MOR^ neurons with PB GABAergic and non-GABAergic neurons. The Cs^+^ based internal solution contained (in mM): CsCl 132, HEPES 10, QX-314 5, MgCl_2_ 2, CaCl_2_ 0.16, EGTA 0.5, Mg-ATP 2, Na-GTP 0.4 (pH = 7.3-7.4, ~295 mOsm). The evoked IPSC was recorded in the presence of the AMPA receptor antagonist NBQX (10 μM), NMDA antagonist (R)-CPP (5 μM), glycine receptor antagonist strychnine (1 μM) and GABA_B_ receptor antagonist CGP55845 (5 μM) in the ACSF with a holding voltage of -70 mV.

To define whether there is local inhibition between GABAergic neurons and *Tacr1^+^* neurons in PB, the Cs^+^ based internal solution contained (in mM): CsCl 132, HEPES 10, QX-314 5, MgCl_2_ 2, CaCl_2_ 0.16, EGTA 0.5, Mg-ATP 2, Na-GTP 0.4 (pH = 7.3-7.4, ~295 mOsm). LED-evoked IPSC was recorded in mCherry-labeled *Tacr1^+^* neurons in in the presence of the AMPA receptor antagonist NBQX (10 μM), NMDA antagonist (R)-CPP (5 μM), glycine receptor antagonist strychnine (1 μM) and GABA_B_ receptor antagonist CGP55845 (5 μM) in the ACSF with a holding voltage of -70 mV. Tetrodotoxin (TTX, 0.5 μM) and 4-aminopyridine (4-AP, 100 μM) were used for further verification of the monosynaptic connection and GABA_A_ receptor antagonist picrotoxin (PTX, 50 μM) was used for the verification of GABA_A_ current.

To identify the recorded neurons, brain slices with the biocytin-filled cells were fixed in 4% PFA at 4 °C overnight. After washed in PBS for 10 min 3 times, the slices were incubated with the streptavidin-405 or streptavidin-488 (Invitrogen) in 0.3% PBST at room temperature for 2 h. After three washes in PBS, the slices were mounted, coverslipped and stored at 4 °C. DAMGO, NBQX, (R)-CPP, strychnine, CGP55845, PTX, and 4-AP were purchased from Tocris Bioscience and TTX was purchased from Hebei Aquatic Product. All other chemicals were purchased from Sigma.

**QUANTIFICATION AND STATISTICAL ANALYSIS**

Statistical analysis was performed using MATLAB 2009a, MATLAB 2013b and Prism 8 (GraphPad Software). The data were analyzed using one-way ANOVA, two-way ANOVA, followed by Bonferroni post hoc analysis, and unpaired or paired student’s *t*-test. All data met the assumptions of the statistical tests used. All data are presented as mean with SEM.

**DATA AND CODE AVAILABILITY**

All fMOST data can be visualized and downloaded from the website https://mouse.digital-brain.cn/projectome/pvh_oxt. All other relevant data and code for this study can be made available by the Lead Contact upon reasonable request.

**SUPPLEMENTARY FIGURES**

**
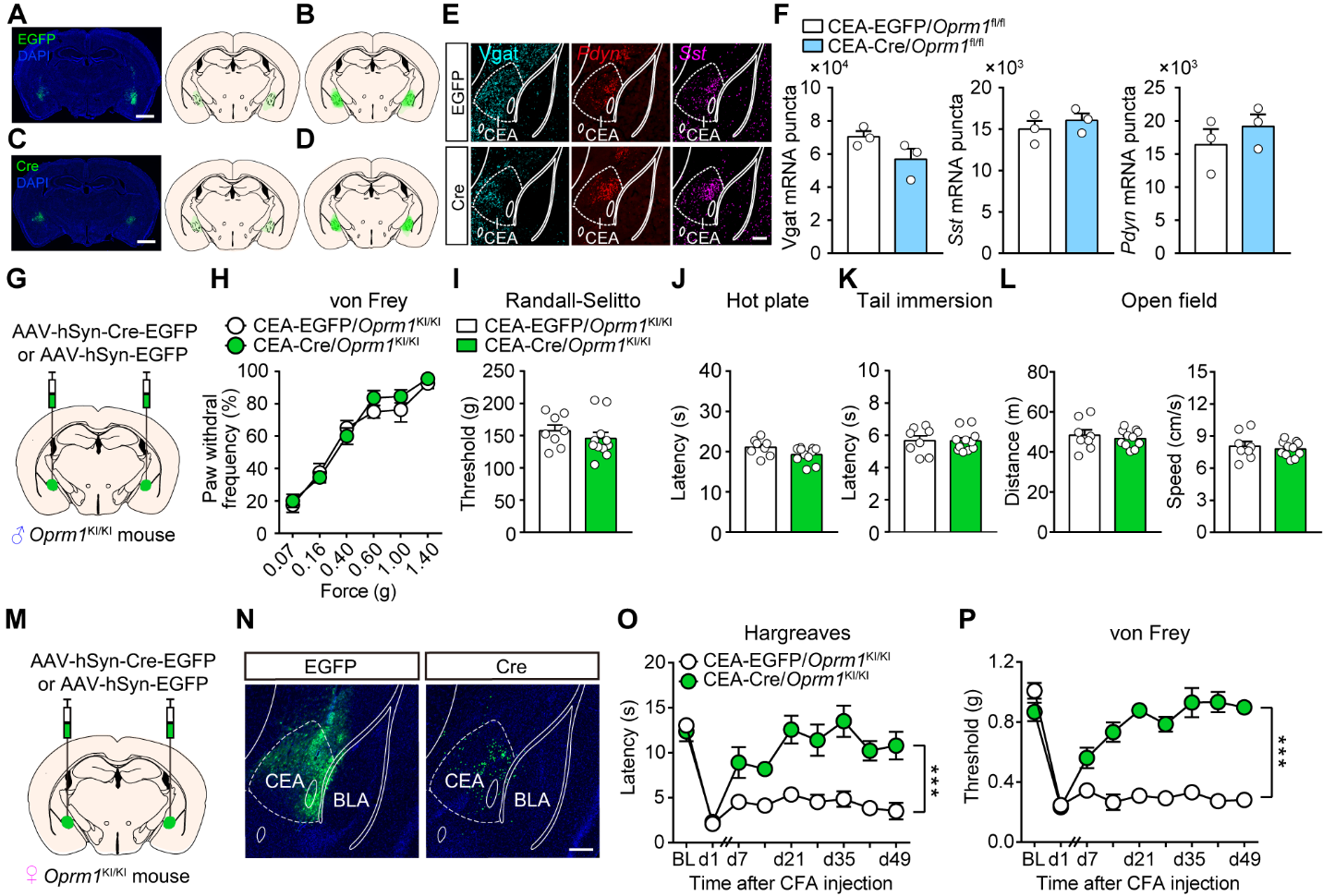
**

Figure S1. Functional role of MORs in the CEA and CFA-induced persistent hyperalgesia. Related to Figure 1.

(A) Left, graphs showing representative images from an *Oprm1*^fl/fl^ mouse bilaterally injected with AAV-hSyn-EGFP virus in the CEA. Scale bar, 1 mm. Right, depiction of virus infection area according to the fluorescent image.

(B) Superimposed depiction of virus infection area for nine male CEA-EGFP/*Oprm1*^fl/fl^ mice.

(C) Left, graphs showing representative images from an *Oprm1*^fl/fl^ mouse bilaterally injected with AAV-hSyn-Cre-EGFP virus in the CEA. Scale bar, 1 mm. Right, depiction of virus infection area according to the fluorescent image.

(D) Superimposed depiction of virus infection area for ten male CEA-Cre/*Oprm1*^fl/fl^ mice.

(E) Graph showing the distribution of Vgat, *Pdyn,* and *Sst* mRNA in the CEA of CEA-EGFP/*Oprm1*^fl/fl^ and CEA-Cre/*Oprm1*^fl/fl^ mice. n = 3-6 mice. Scale bar, 200 μm.

(F) Vgat^+^, *Pdyn*^+^ and *Sst*^+^ mRNA puncta in the CEA. n = 3 mice. Student’s unpaired *t* test.

(G) Schematic showing bilateral injection of AAV-hSyn-Cre-EGFP or AAV-hSyn-EGFP virus into the CEA of male *Oprm1*^KI/KI^ mouse.

(H and I) Effects of *Oprm1* re-expression in the CEA on mechanical nociception tested with von Frey (H) and Randall-Selitto (I) tests. n = 8-11 mice. Two-way ANOVA followed by Bonferroni correction (H) and Student’s unpaired *t* test (I).

(J and K) Effects of *Oprm1* re-expression in the CEA on thermal nociception tested with tail immersion (48 °C, J) and hot plate (52 °C, K) tests. n = 8-11 mice. Student’s unpaired *t* test.

(L) Effects of *Oprm1* re-expression in the CEA on locomotor activity in open field test. n =8-11 mice. Student’s unpaired *t* test.

(M) Schematic showing bilateral injection of AAV-hSyn-Cre-EGFP or AAV-hSyn-EGFP virus into the CEA of female *Oprm1*^KI/KI^ mouse.

(N) Graph showing the virus expression in the CEA of female *Oprm1*^KI/KI^ mouse. Scale bar, 200 μm.

(O and P) Thermal (O) and mechanical (P) hyperalgesia on CFA-induced persistent hyperalgesia in ipsilateral hindpaws of female *Oprm1*^KI/KI^ mice. n = 5 mice for each group. BL, baseline, indicating the basal nociceptive sensitivity before CFA injection. d1, day 1. Two-way ANOVA followed by Bonferroni correction.

****P* < 0.001. Data are presented as mean ± SEM.


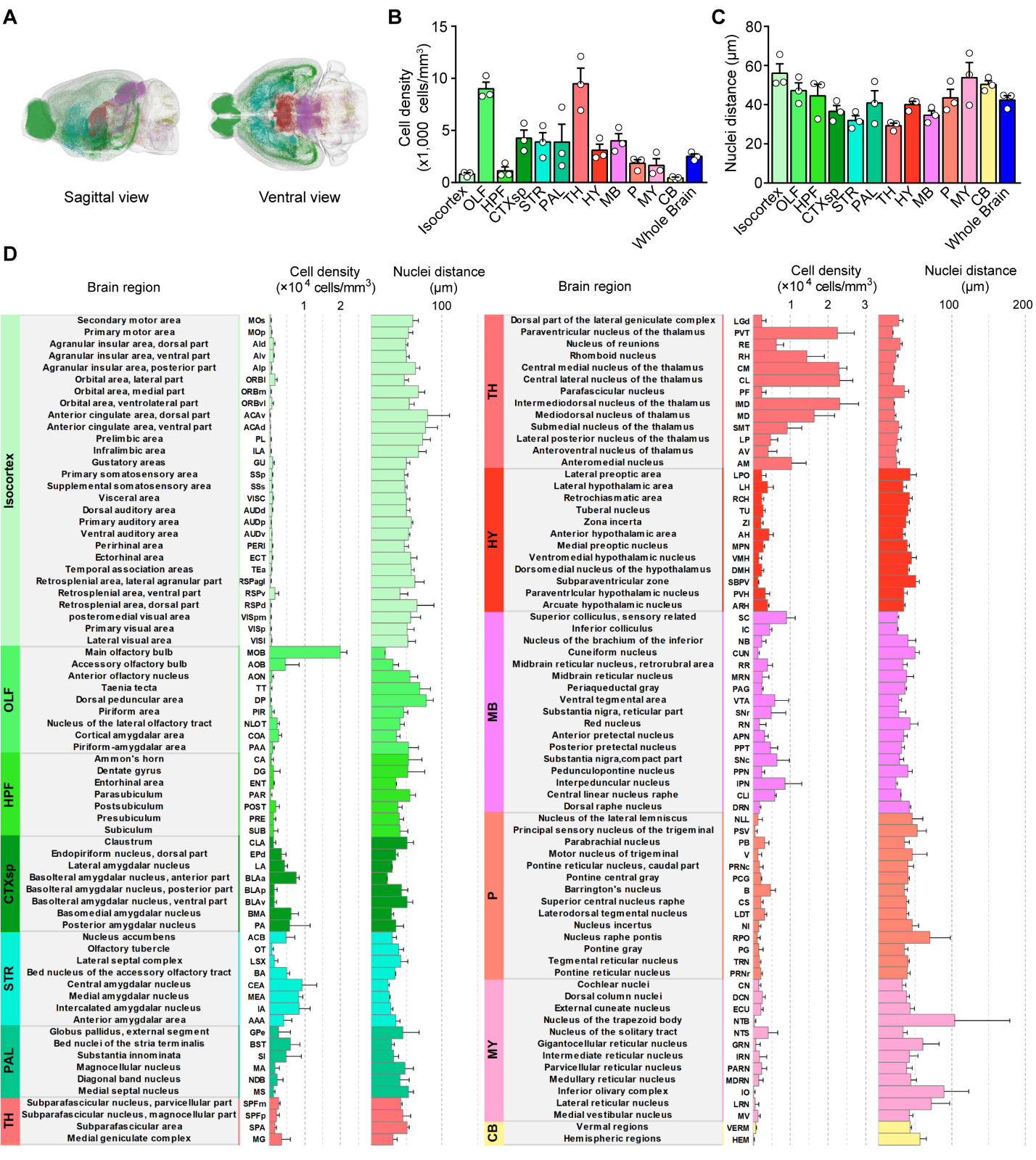


**Figure S2. Distribution of MOR^+^ neurons in the mouse brain. Related to Figure 2.** (A) Recognized position of tdTomato-labeled MOR^+^ neurons in a male MOR-iCreER^T2^ × Ai9 mouse brain. Different colors of points correspond to separated brain regions in (B-D). Sagittal view (left), ventral view (right).

(B) Cell density of tdTomato-labeled MOR^+^ neurons in brain regions.

(C) Nuclei distance of tdTomato-labeled MOR^+^ neurons in brain regions. Circles represent individual counts of sections from three mice. OLF, olfactory areas; HPF, hippocampal formation; CTXsp, cortical subplate; STR, striatum; PAL, pallidum; TH, thalamus; HY, hypothalamus; MB, midbrain; P, pons; MY, medulla; CB, cerebellum.

(D) Cell density and nuclei distance of tdTomato-labeled MOR^+^ neurons in the representative subregions of the above brain regions. n = 3 mice.

Data are presented as mean ± SEM.


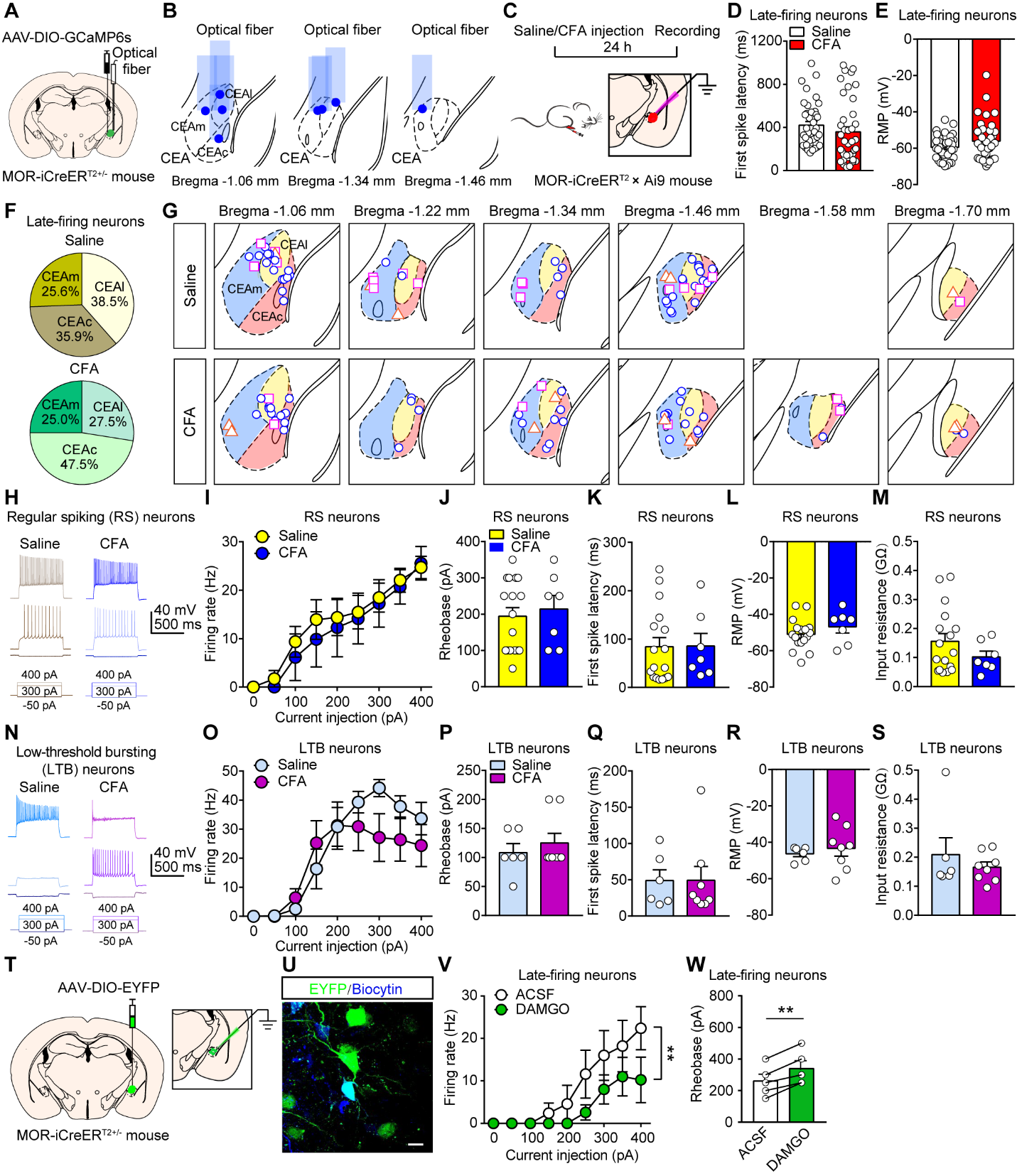


**Figure S3. The excitability of CEA^MOR^ neurons increased during persistent hyperalgesia. Related to Figure 2.**

(A) Schematic diagram showing stereotaxic injection and optical fiber implantation above the right CEA of male MOR-iCreER^T2+/-^ moue.

(B) Location of the tips of optical fibers above the right CEA. n = 8 mice.

(C) Schematic showing the electrophysiological recording on tdTomato-labeled MOR^+^ neurons in the ipsilateral CEA of male MOR-iCreER^T2^ × Ai9 mouse, which was injected with CFA (50%, 20 μl) or saline 1 day ago.

(D and E) Summary data showing the first spike latency (D) and resting membrane potential (RMP, E) of recorded “late-firing” CEA^MOR^ neurons in ACSF with NBQX (10 μM), CPP (5 μM), STR (2 μM), CGP (5 μM) and PTX (50 μM). Saline group: n = 39 neurons from 3 mice; CFA group: n = 40 neurons from 5 mice. Student’s unpaired *t* test.

(F) The percentage of the recorded CEA^MOR^ neurons with late firing pattern. Saline group: n = 39 neurons from 3 mice; CFA group: n = 40 neurons from 5 mice.

(G) Distribution of recorded CEA^MOR^ neurons in CEA subregions. Saline group: n = 62 neurons from 3 mice; CFA group: n = 55 neurons from 5 mice. “Late-firing”: blue circle; “regular spiking”: pink square; “low-threshold bursting”: orange triangle. CEAl: light yellow; CEAc: light red; CEAm, light blue.

(H) Representative traces of currents evoked by current injection recorded for “regular spiking” CEA^MOR^ neurons.

(I-M) Summary data showing the firing rates (I), rheobase values (J), first spike latency (K), resting membrane potential (RMP, L), and input resistance (M) for “regular spiking” CEA^MOR^ neurons. Saline group: n = 17 neurons from 3 mice; CFA group: n = 7 neurons from 5 mice. Two-way ANOVA followed by Bonferroni correction (I) and Student’s unpaired *t* test (J-M).

(N) Representative traces of currents evoked by current injection recorded for “low-threshold bursting” CEA^MOR^ neurons.

(O-S) Summary data showing the firing rates (O), rheobase values (P), first spike latency (Q), resting membrane potential (RMP, R), and input resistance (S) for “low-threshold bursting” CEA^MOR^ neurons. Saline group: n = 6 neurons from 3 mice; CFA group: n = 8 neurons from 5 mice. Two-way ANOVA followed by Bonferroni correction (O) and Student’s unpaired *t* test (P-S).

(T) Schematic showing injection of AAV-Ef1α-DIO-EYFP virus into the right CEA of male MOR-iCreER^T2+/-^ mouse and the whole-cell patch clamp recording was performed on EYFP-labeled CEA^MOR^ neurons.

(U) Post hoc staining of a recorded EYFP-labeled CEA^MOR^ neuron (green) with biocytin (blue). Scale bar, 10 μm.

(V and W) Summary data showing the firing rates (V) and rheobase values (W) for EYFP-labeled CEA^MOR^ neurons. n = 5 neurons from 2 mice. Two-way ANOVA followed by Bonferroni correction (V) and Student’s paired *t* test (W).

***P* < 0.01. Data are presented as mean ± SEM.

**
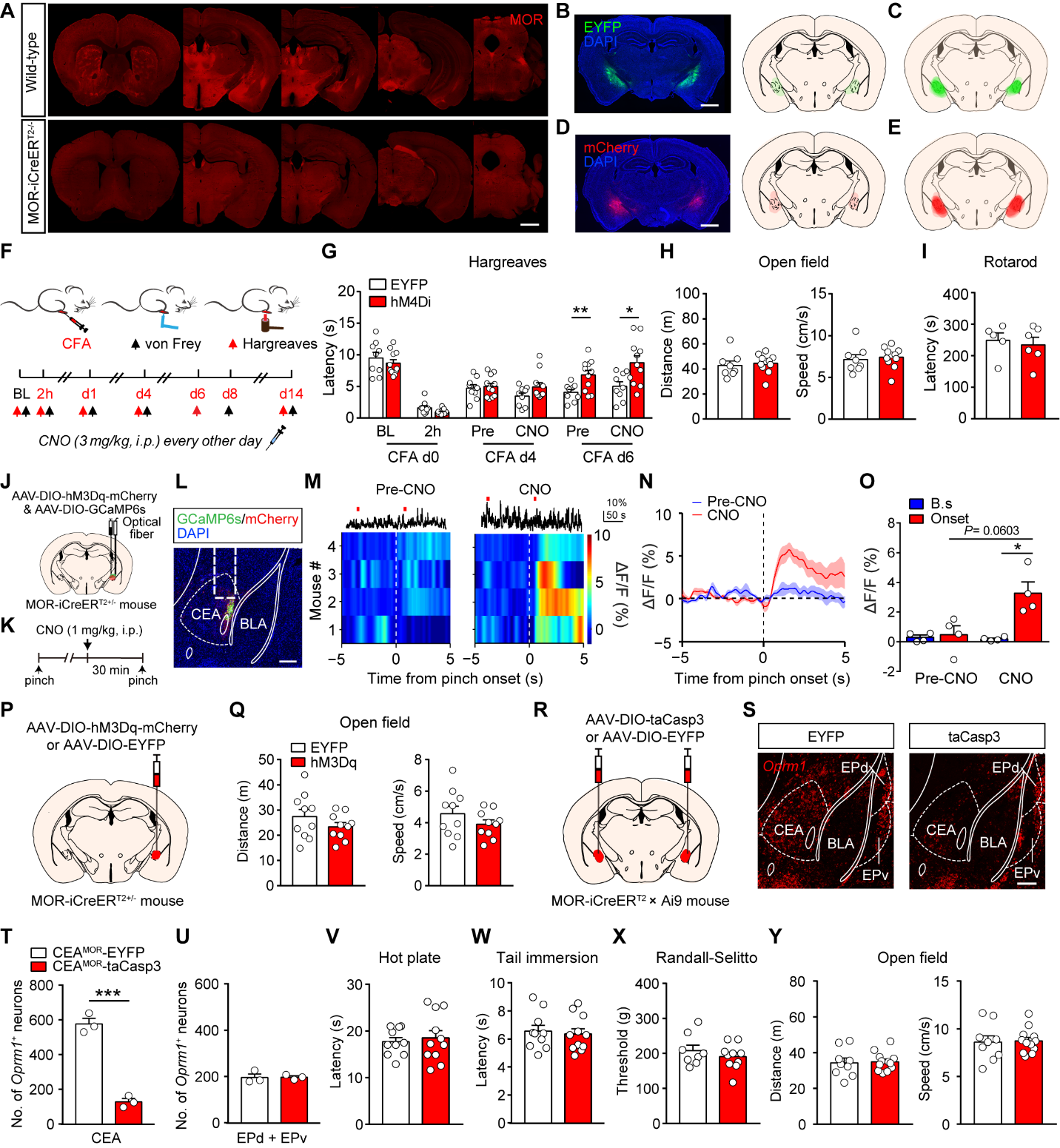
**

**Figure S4. Functional role of CEA^MOR^ neurons in modulation of nociception. Related to Figure 3.**

(A) Images showing the immunostaining of MOR in brain sections of male MOR-iCreER^T2-/-^ and wild-type mice. Scale bar, 1 mm.

(B) Left, a representative image from a male MOR-iCreER^T2-/-^ mouse bilaterally injected with AAV-Ef1α-DIO-EYFP virus in the CEA. Scale bar, 1 mm. Right, depiction of virus infection area according to the fluorescent image.

(C) Superimposed depiction of virus infection area for mice in EYFP group. n = 9 mice.

(D) Left, a representative image from a male MOR-iCreER^T2-/-^ mouse bilaterally injected with AAV-hSyn-DIO-hM4Di-mCherry virus in the CEA. Scale bar, 1 mm. Right, depiction of virus infection area according to the fluorescent image.

(E) Superimposed depiction of virus infection area for mice in hM4Di group. n = 11 mice.

(F) Schematic diagram showing design of the experiment and timeline of the behavioral tests in CFA-induced persistent hyperalgesia. BL, baseline, indicating the basal nociceptive sensitivity before CFA injection. d1, day 1.

(G) Thermal hyperalgesia on CFA-induced hyperalgesia in ipsilateral hindpaws of male MOR-iCreER^T2-/-^ mice before (Pre) and 30 min after CNO (3 mg/kg, i.p.) injection on specific days after CFA injection. n = 9-11 mice. BL, baseline, indicating the basal nociceptive sensitivity before CFA injection. d0, day 0. Two-way ANOVA followed by Bonferroni correction.

(H) Effect of pharmacogenetic inhibition of CEA^MOR^ neurons on locomotor activity in open field test. n = 8-11 mice. Student’s unpaired *t* test.

(I) Effect of pharmacogenetic inhibition of CEA^MOR^ neurons on motor ability in rotarod test. n = 5-6 mice. Student’s unpaired *t* test.

(J) Schematic showing injection of mixed AAV-hSyn-DIO-hM3Dq-mCherry and AAV-Ef1α-DIO-GCaMP6s viruses into the right CEA of male MOR-iCreER^T2+/-^ mouse, followed by implantation of an optical fiber.

(K) Schematic diagram showing design of the experiment and timeline.

(L) A representative imaging showing the expression of GCaMP6s and hM3Dq as indicated by mCherry. Scale bar, 200 μm.

(M) Heat map showing response of CEA^MOR^ neurons to paw pinch before and after CNO injection. Each red bar represents a pinch event. Each row represents the response of one mouse.

(N) Averaged calcium response of CEA^MOR^ neurons to paw pinch before and after CNO injection.

(O) Comparison of the averaged fluorescence signal change during paw pinch baseline (-5-0 s, B.s) and onset period (0-5 s) in each session. n = 4 mice. Two-way ANOVA followed by Bonferroni correction.

(P) Schematic showing injection of AAV-hSyn-DIO-hM3Dq-mCherry or AAV-Ef1α-DIO-EYFP virus into the right CEA of male MOR-iCreER^T2+/-^ mouse.

(Q) Effects of CEA^MOR^ neuron activation on locomotor activity in open field test. n = 10 mice for each group. Student’s unpaired *t* test.

(R) Schematic showing bilateral injection of AAV-CAG-DIO-taCasp3 or AAV-Ef1α-DIO-EYFP virus into the CEA of male MOR-iCreER^T2^ × Ai9 mouse.

(S) RNAscope data showing the *Oprm1*^+^ neurons in the CEA of MOR-iCreER^T2^ × Ai9 mouse. Scale bar, 200 μm.

(T-U) Number of *Oprm1^+^* neurons in CEA (T) or EPd+EPv (U) in MOR-iCreER^T2^ × Ai9 mice. n = 3 mice. Student’s unpaired *t* test. EPd, Endopiriform nucleus, dorsal part; EPv, Endopiriform nucleus, ventral part.

(V-X) Effects of CEA^MOR^ neuron ablation on nociceptive behavioral tests with hot plate (52 °C, V), tail immersion (48 °C, W), Randall-Selitto (X) tests. n = 10-11 mice. Student’s unpaired *t* test (Y) Effects of CEA^MOR^ neuron ablation on locomotor activity in open field test. n = 9-12 mice. Student’s unpaired *t* test.

**P* < 0.05, ***P* < 0.01, ****P* < 0.001. Data are presented as mean ± SEM.

**
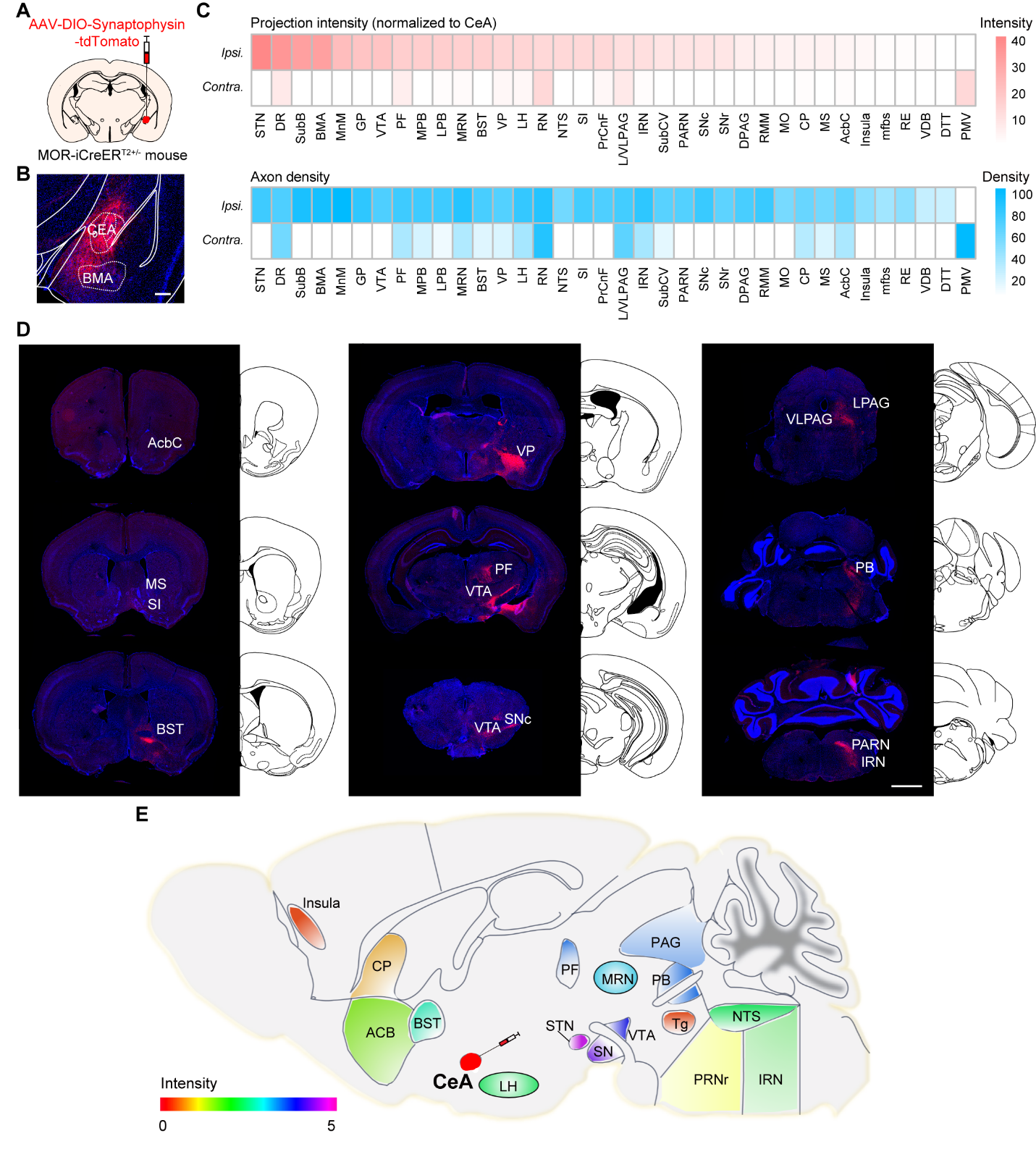
**

**Figure S5. Whole-brain mapping of axonal projections from CEA^MOR^ neurons.** **Related to Figure 4.**

(A) Schematic showing the anterograde axonal AAV tracing used to determine projection of CEA^MOR^ neurons.

(B) Graph showing virus expression in CEA and BMA (basomedial amygdalar nucleus). Scale bar, 200 μm.

(C) Heatmap showing the ipsilateral projection from CEA^MOR^ neurons in representative brain regions of male MOR-iCreER^T2+/-^ mice. Top, Lightsalmon pink to White Color bar indicates projection intensity level (ranging from 10 to 40). Bottom, Blue to White Color bar indicates axonal projection distribution level (ranging from 20 to 100). Each column represents averaged values from 3 mice.

(D) Representative images from a MOR-iCreER^T2+/-^ mouse showing whole-brain mapping of axonal projections. Scale bar, 1 mm.

(E) An overview of the whole-brain axon projections of CEA^MOR^ neurons. STN, subthalamic nucleus; DR, dorsal raphe nucleus; SubB, subbrachial nucleus; BMA, basomedial amygdalar nucleus; MnM, medial mammillary nucleus, median part; GP, globus pallidus; VTA, ventral tegmental area; PF, parafascicular thalamic nucleus; MPB, medial parabrachial nucleus; LPB, lateral parabrachial nucleus; SNL, substantia, lateral part; MRN, midbrain reticular nucleus; BST, bed nucleus stria terminalis; PALv, Pallidum, ventral region; LH, lateral hypothalamic area; NTS, nucleus of the solitary tract; SI, substantia innominata; PrCnF, precuneiform area; L/VLPAG, lateral and ventrolateral periaqueductal gray; IRN, intermediate reticular nucleus; SubCV, subcoeruleus nucleus, ventral part; PARN, parvicellular reticular nucleus; SNc, substantia nigra, compact; SNr, substantia nigra, reticular part; DPAG, dorsal periaqueductal gray; RMM, retromammillary nucleus, medial part; MO, somatomotor areas, CP, caudoputamen; MS, medial septal nucleus; AcbC, nucleus accumbens, core; Insular, insular cortex; mfbs, medial forebrain bundle system; RE, nucleus of reuniens; VDB, nucleus of the vertical limb of the diagonal band; DTT, dorsal tenia tecta; RN, reticular nucleus of the thalamus; PMV, premammillary nucleus, ventral part; ACB, nucleus accumbens; SN, substantia nigra; Tg, pedunculotegmental nucleus (PPN) and laterodorsal tegmental nucleus (LDT); PRNr, pontine reticular nucleus. The value of projection intensity normalized against injection site. n = 3 mice.


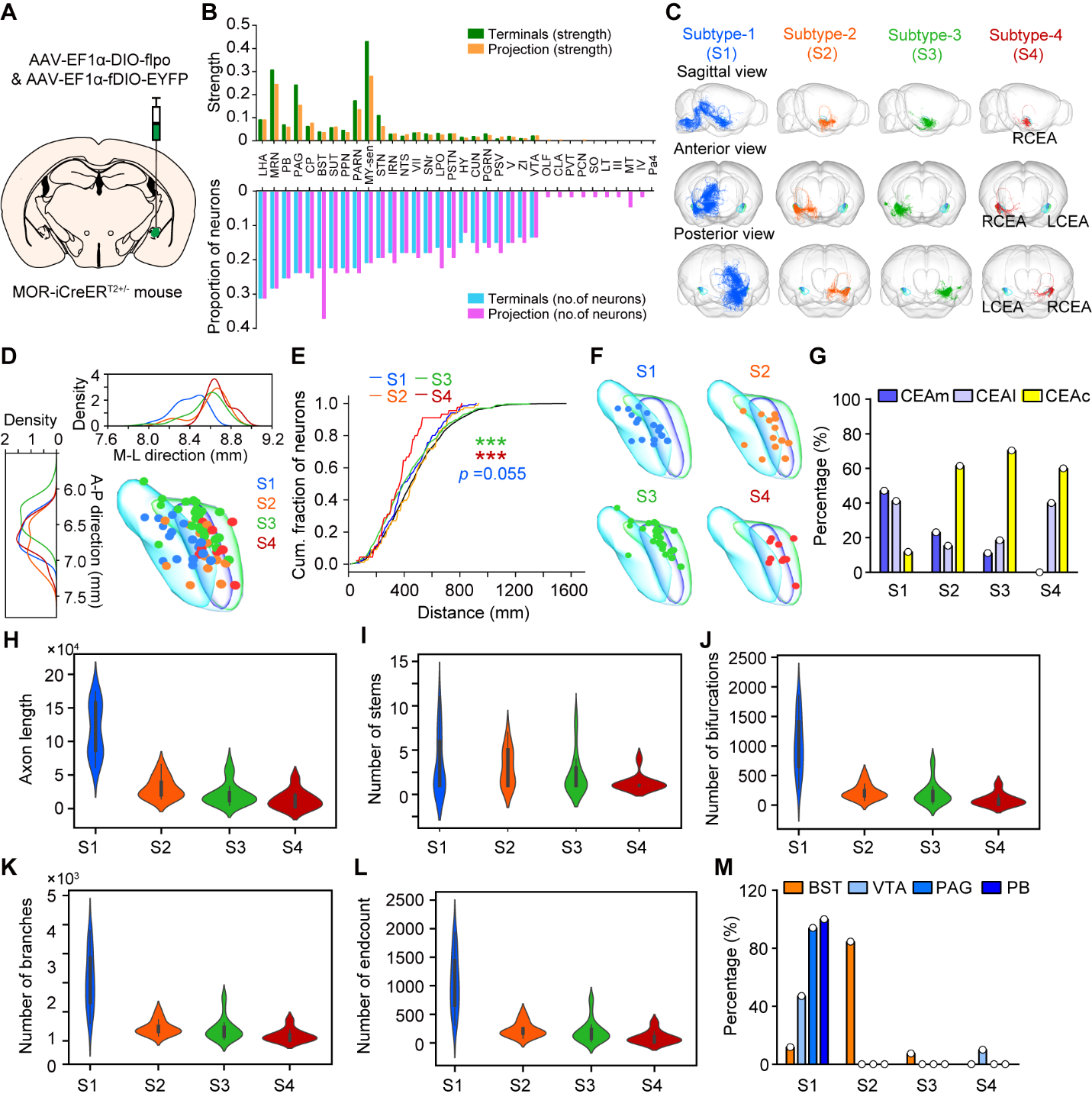


**Figure S6. Single cell projectome of CEA^MOR^ neurons. Related to Figure 4.**

(A) Schematic showing the sparse labeling strategy by injection of sparse labeling virus into the right CEA of male MOR-iCreER^T2+/-^ mouse.

(B) Top, total axon length (orange) of all 67 neurons normalized by maximal axon length of sub-region (CEA, which was not exhibited) and number of terminals (green) of all 67 neurons normalized by maximal number of terminals of sub-region (CEA). Bottom, proportion of neurons that sent axons (carmine) or had terminals (blue) in each region. Abbreviations for brain regions were listed in Table S1.

(C) Sagittal, anterior and posterior views of 4 identified subtypes of CEA^MOR^ neurons. Subtype-1: blue, subtype-2: orange, subtype-3: green, subtype-4: red. Blueish region: LCEA, left CEA; RCEA, right CEA. n = 67 neurons from 4 mice.

(D) Topographic assemble of somata of CEA^MOR^ neurons. Top, histogram of location of soma along medial-lateral direction. Left, histogram of location of soma along anterior-posterior direction. Middle, soma location. Color was coded according to the subtypes division.

(E) Cumulative distribution of soma location of neurons in each subtype.

(F) Soma location of 4 subtypes neurons in 3 subregions of CEA. CEAm, medial part of CEA; CEAl, lateral part of CEA; CEAc, capsular part of CEA.

(G) Percentage of neurons distributed in 3 subregions of CEA.

(H-L) Morphological parameters of neurons in each subtype: axon length (H), number of stems (I), number of bifurcations (J), number of branches (K), and number of endcount (L).

(M) Percentage of neurons in each subtype that projected to BST, VTA, PAG and PB.


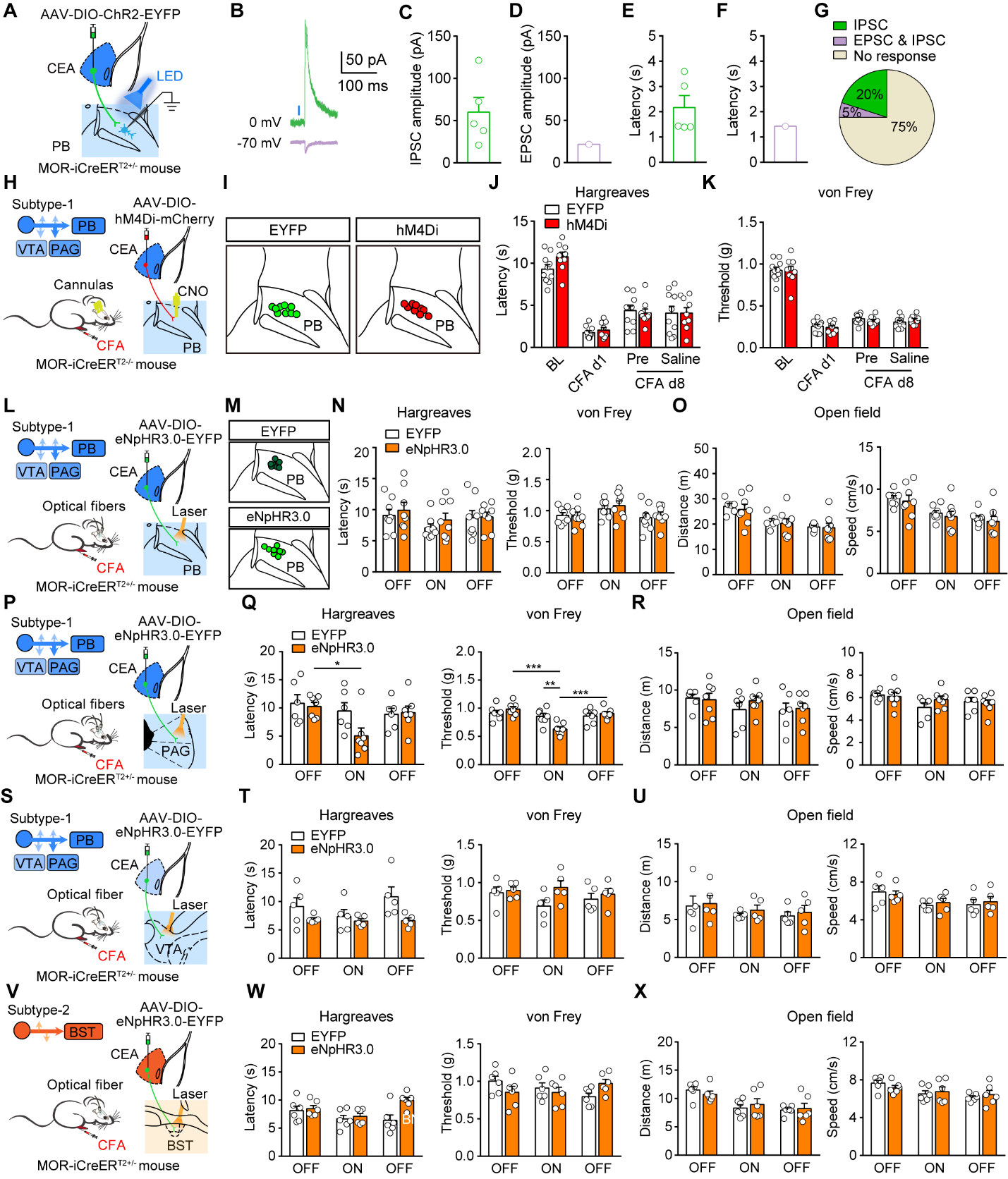


**Figure S7. Functional role of the projection from CEA^MOR^ neurons to PB in CFA-induced persistent hyperalgesia. Related to Figure 5.**

(A) Schematic showing injection of AAV-Ef1α-DIO-ChR2-EYFP into the right CEA of male MOR-CreER^T2+/-^ mouse.

(B) Representative light-evoked IPSC and EPSC recorded from PB neurons. Blue bar, LED stimulation (475 nm; 1 ms).

(C and D) A summary showing the amplitudes of IPSCs (5/20 neurons, C) and EPSC (1/20 neurons, D) in PB neurons induced by the photoactivation of ChR2^+^ fibers from CEA^MOR^ neurons.

(E and F) A summary showing the latencies of IPSCs (E) and EPSCs (F).

(G) Summary of the connection rate. n = 20 neurons from two mice.

(H) Schematic showing injection of AAV-hSyn-DIO-hM4Di-mCherry virus into the right CEA, and implantation of cannula above the right PB of male MOR-iCreER^T2-/-^ mouse.

(I) Distribution of the cannula tip locations in the PB of MOR-iCreER^T2-/-^ mice. n = 9-10 mice.

(J and K) Thermal (J) and mechanical (K) hyperalgesia in ipsilateral hind paws before (Pre) and 30 min after saline injection. n = 9-10 mice. BL, baseline, indicating the basal nociceptive sensitivity before CFA injection. d1, day 1.

(L) Schematic showing bilateral injection of AAV-Ef1α-DIO-eNpHR3.0-EYFP virus into the CEA, and implantation of optical fibers above the PB of male MOR-iCreER^T2+/-^ mouse.

(M) Distribution of the optical fiber tip locations in the PB of MOR-iCreER^T2+/-^ mice. n = 8 mice for each group.

(N) Effect of optogenetic suppression of the projection from CEA^MOR^ neurons to PB on basal nociceptive sensitivity tested by Hargreaves (left) and von Frey (right) tests on MOR-iCreER^T2+/-^ mice before CFA application. n = 8 mice for each group.

(O) Effects of optogenetic suppression of the projection from CEA^MOR^ neurons to PB on locomotor activity in open field test. n = 8 mice for each group.

(P) Schematic showing injection of AAV-Ef1α-DIO-eNpHR3.0-EYFP virus into the right CEA, and implantation of optical fiber above the right PAG of male MOR-iCreER^T2+/-^ mouse.

(Q) Effect of optogenetic suppression of the ipsilateral projection from CEA^MOR^ neurons to PAG on basal nociceptive sensitivity tested by Hargreaves (left) and von Frey (right) tests on MOR-iCreER^T2+/-^ mice before CFA application. n = 6-7 mice.

(R) Effects of optogenetic suppression of the ipsilateral projection from CEA^MOR^ neurons to PAG on locomotor activity in open field test. n = 6-7 mice.

(S) Schematic showing injection of AAV-Ef1α-DIO-eNpHR3.0-EYFP virus into the right CEA, and implantation of optical fiber above the right VTA of male MOR-iCreER^T2+/-^ mouse.

(T) Effect of optogenetic suppression of the ipsilateral projection from CEA^MOR^ neurons to VTA on basal nociceptive sensitivity tested by Hargreaves (left) and von Frey (right) tests on MOR-iCreER^T2+/-^ mice before CFA application. n = 5 mice for each group.

(U) Effects of optogenetic suppression of the ipsilateral projection from CEA^MOR^ neurons to VTA on locomotor activity in open field test. n = 5 mice for each group.

(V) Schematic showing injection of AAV-Ef1α-DIO-eNpHR3.0-EYFP virus into the right CEA, and implantation of optical fiber above the right BST of male MOR-iCreER^T2+/-^ mouse.

(W) Effect of optogenetic suppression of the ipsilateral projection from CEA^MOR^ neurons to BST on basal nociceptive sensitivity tested by Hargreaves ((left) and von Frey (right) tests on MOR-iCreER^T2+/-^ mice before CFA application. n = 6 mice for each group.

(X) Effects of optogenetic suppression of the ipsilateral projection from CEA^MOR^ neurons to BST on locomotor activity in open field test. n = 6 mice for each group.

**P* < 0.05, ***P* < 0.01, ****P* < 0.001 with two-way ANOVA followed by Bonferroni correction. Data are presented as mean ± SEM.


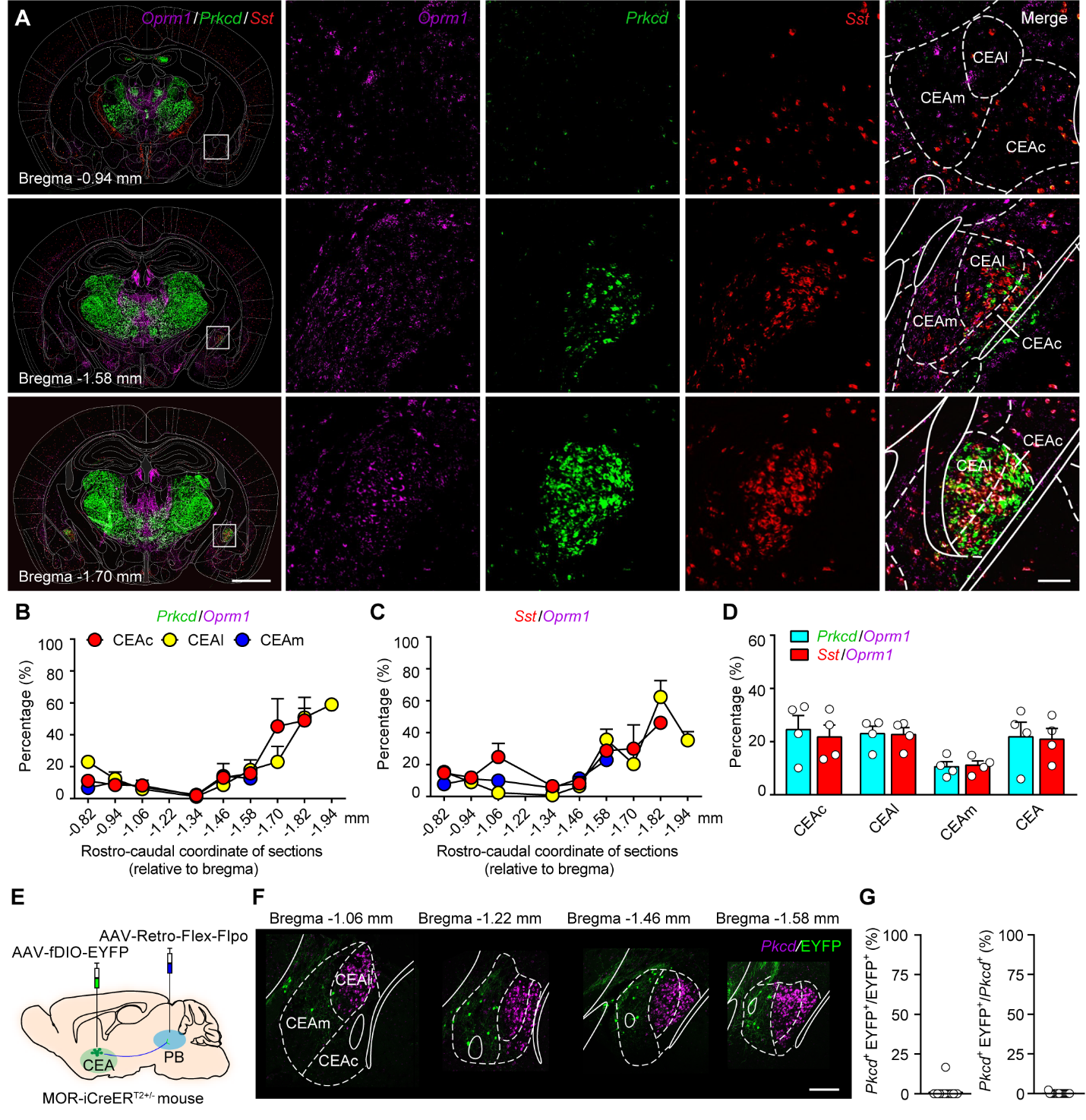


**Figure S8. Distribution of different subsets of neurons in the CEA.** **Related to Figure 5.**

(A) Multiple in situ hybridization in male wild-type mice shows the distribution of *Oprm1* in *Prkcd*^+^ and *Sst*^+^ neurons using RNAscope assay. Scale bars, 1 mm (left), 100 μm (right). (B and C) Rostro-caudal distribution of *Prkcd*^+^ and *Sst*^+^ neurons in *Oprm1*^+^ neurons in the CEA.

(D) Percentage *Prkcd*^+^ and *Sst*^+^ neurons in *Oprm1*^+^ neurons in the CEA and subregions (CEAc, CEAl and CEAm). n = 4 mice.

(E) Schematic showing viral injection into the right PB and CEA of male MOR-iCreER^T2+/-^ mouse.

(F) The distribution of EYFP-labeled PB-projecting CEA^MOR^ neurons and *Pkcd^+^* neurons in the CEA. Scale bar, 200 μm.

(G) Percentage of co-localized neurons in EYFP-labeled PB-projecting CEA^MOR^ neurons and *Pkcd^+^* neurons in the CEA. n = 17 sections from 3 mice.

Data are presented as mean ± SEM.


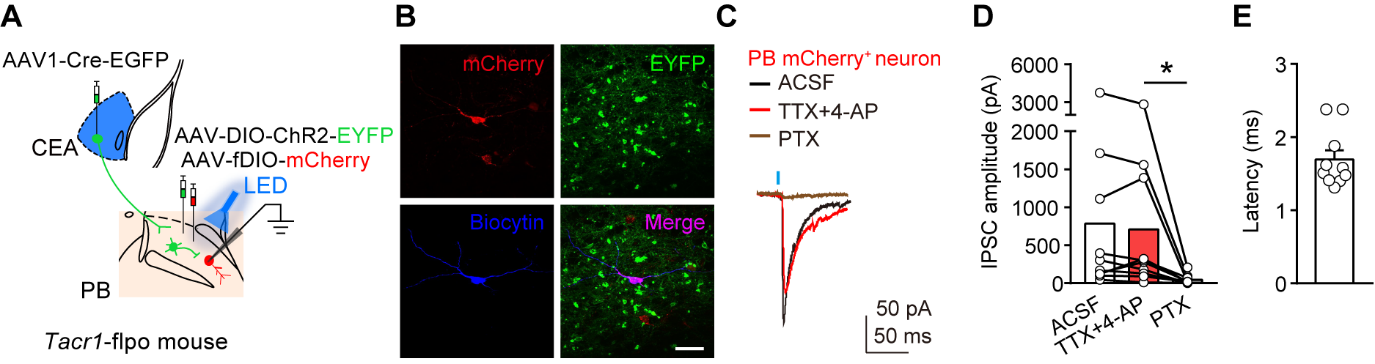


**Figure S9. Functional synaptic connection between PB neurons receiving CEA projection and PB*^Tacr1^* neurons. Related to Figure 6.**

(A) Schematic showing viral injection into the right CEA and PB and electrophysiological recording of PB neurons of male *Tacr1*-flpo mouse.

(B) Post hoc staining of recorded mCherry-labeled PB*^Tacr1^* neurons for biocytin (blue) and EYFP^+^ fibers. Scale bar, 50 μm.

(C) The light-evoked IPSCs recorded at -70 mV in a mCherry^+^ PB (PB*^Tacr1^*) neuron in the present of TTX and 4-AP. Blue bars indicate photostimulation (473 nm, 1 ms).

(D and E) The light (473 nm, 1 ms, 10 Hz)-evoked IPSCs (D) and latency (E) recorded at -70 mV in mCherry^+^ neurons. n = 10 neurons from 4 mice. Student’s paired *t* test.

**P* < 0.05. Data are presented as mean ± SEM.

**SUPPLEMENTARY MOVIE**

Movie S1. 3D visualization of tdTomato-labeled image dataset of whole MOR-iCreER^T2^ × Ai9 mouse brain. Original dataset was registered to the Allen CCFv3 and down-sampled at the voxel size of 3 μm × 3 μm × 2 μm for 3D volume rendering due to the restriction of graphic card performance.

Movie S2. 3D distribution of automatically detected tdTomato-labeled MOR^+^ neurons in the CEA of male MOR-iCreER^T2^ × Ai9 mouse brain. The three sub-structures are shown in different colors for location reference. CEAc, red; CEAl, yellow; CEAm, blue.

**SUPPLEMENTARY TABLE**

**Table S1. Abbreviations for brain regions. Related to Figures 4 and S6.**

| Abbreviation | Full name |
| --- | --- |
| AAA | Anterior amygdalar area |
| ACB | Nucleus accumbens |
| BLA | Basolateral amygdalar nucleus |
| BMA | Basomedial amygdalar nucleus |
| BST | Bed nuclei of the stria terminalis |
| CB | Cerebellum |
| CEA | Central amygdalar nucleus |
| CLA | Claustrum |
| CP | Caudoputamen |
| CTXsp | Cortical subplate |
| CUN | Cuneiform nucleus |
| DR | Dorsal nucleus raphe |
| EPd | Endopiriform nucleus, dorsal part |
| EPv | Endopiriform nucleus, ventral part |
| FS | Fundus of striatum |
| GRN | Gigantocellular reticular nucleus |
| HPF | Hippocampal formation |
| HY | Hypothalamus |
| IA | Intercalated amygdalar nucleus |
| III | Oculomotor nucleus |
| IRN | Intermediate reticular nucleus |
| IV | trochlear |
| LHA | Lateral hypothalamic area |
| LIN | Linear nucleus of the medulla |
| LPO | Lateral preoptic area |
| LT | Lateral terminal nucleus of the accessory optic tract |
| MB | Midbrain |
| MDRN | Medullary reticular nucleus |
| MEA | Medial amygdalar nucleus |
| MRN | Midbrain reticular nucleus |
| MT | Medial terminal nucleus of the accessory optic tract |
| MY | Medulla |
| MY-sen | Medulla, sensory related |
| NTS | Nucleus of the solitary tract |
| OLF | Olfactory areas |
| P | Pons |
| P5 | Peritrigeminal zone |
| Pa4 | Paratrochlear nucleus |
| PAG | Periaqueductal gray |
| PAL | Pallidum |
| PARN | Parvicellular reticular nucleus |
| PB | Parabrachial nucleus |
| PCN | Paracentral nucleus |
| PGRN | Paragigantocellular reticular nucleus |
| PoT | Posterior triangular thalamic nucleus |
| PPN | Pedunculopontine nucleus |
| PRNc | Pontine reticular nucleus, caudal part |
| PSTN | Parasubthalamic nucleus |
| PSV | Principal sensory nucleus of the trigeminal |
| PVT | Paraventricular nucleus of the thalamus |
| RR | Midbrain reticular nucleus, retrorubral area |
| SCm | Superior colliculus, motor related |
| SI | Substantia innominata |
| SNc | Substantia nigra, compact part |
| SNr | Substantia nigra, reticular part |
| SO | Supraoptic nucleus |
| SOC | Superior olivary complex |
| SPF | Subparafascicular nucleus |
| STN | Subthalamic nucleus |
| STR | Striatum |
| SUT | Supratrigeminal nucleus |
| TH | Thalamus |
| V | Motor nucleus of trigeminal |
| VII | Facial motor nucleus |
| VTA | Ventral tegmental area |
| XII | Hypoglossal nucleus |
| ZI | Zona incerta |

**SUPPLEMENTARY REFERENCE**

Chaplan, S.R., Bach, F.W., Pogrel, J.W., Chung, J.M., and Yaksh, T.L. (1994). Quantitative assessment of tactile allodynia in the rat paw. J Neurosci Methods *53*, 55-63.

Deng, J., Zhou, H., Lin, J.K., Shen, Z.X., Chen, W.Z., Wang, L.H., Li, Q., Mu, D., Wei, Y.C., Xu, X.H.*, et al.* (2020). The Parabrachial Nucleus Directly Channels Spinal Nociceptive Signals to the Intralaminar Thalamic Nuclei, but Not the Amygdala. Neuron *107*, 909-923 e906.

Dou, Y., Xia, J., Gao, R., Gao, X., Munoz, F.M., Wei, D., Tian, Y., Barrett, J.E., Ajit, S., Meucci, O.*, et al.* (2018). Orai1 Plays a Crucial Role in Central Sensitization by Modulating Neuronal Excitability. J Neurosci *38*, 887-900.

Gao, L., Liu, S., Gou, L., Hu, Y., Liu, Y., Deng, L., Ma, D., Wang, H., Yang, Q., Chen, Z.*, et al.* (2022). Single-neuron projectome of mouse prefrontal cortex. Nat Neurosci *25*, 515-529.

Gong, H., Xu, D., Yuan, J., Li, X., Guo, C., Peng, J., Li, Y., Schwarz, L.A., Li, A., Hu, B.*, et al.* (2016). High-throughput dual-colour precision imaging for brain-wide connectome with cytoarchitectonic landmarks at the cellular level. Nat Commun *7*, 12142.

Hu, H.J., Carrasquillo, Y., Karim, F., Jung, W.E., Nerbonne, J.M., Schwarz, T.L., and Gereau, R.W.t. (2006). The kv4.2 potassium channel subunit is required for pain plasticity. Neuron *50*, 89-100.

Mu, D., Deng, J., Liu, K.F., Wu, Z.Y., Shi, Y.F., Guo, W.M., Mao, Q.Q., Liu, X.J., Li, H., and Sun, Y.G. (2017). A central neural circuit for itch sensation. Science *357*, 695-699.

Ni, H., Tan, C., Feng, Z., Chen, S., Zhang, Z., Li, W., Guan, Y., Gong, H., Luo, Q., and Li, A. (2020). A Robust Image Registration Interface for Large Volume Brain Atlas. Sci Rep *10*, 2139.

Quan, T., Zheng, T., Yang, Z., Ding, W., Li, S., Li, J., Zhou, H., Luo, Q., Gong, H., and Zeng, S. (2013). NeuroGPS: automated localization of neurons for brain circuits using L1 minimization model. Sci Rep *3*, 1414.

Sun, P., Jin, S., Tao, S., Wang, J., Li, A., Li, N., Wu, Y., Kuang, J., Liu, Y., Wang, L.*, et al.* (2020). Recombinase system-dependent copackaging strategy for highly efficient neurocircuit tracing. bioRxiv.

Wang, Q., Ding, S.L., Li, Y., Royall, J., Feng, D., Lesnar, P., Graddis, N., Naeemi, M., Facer, B., Ho, A.*, et al.* (2020). The Allen Mouse Brain Common Coordinate Framework: A 3D Reference Atlas. Cell *181*, 936-953 e920.

Zhang, C., Yan, C., Ren, M., Li, A., Quan, T., Gong, H., and Yuan, J. (2017). A platform for stereological quantitative analysis of the brain-wide distribution of type-specific neurons. Sci Rep *7*, 14334.

Zhang, X.Y., Dou, Y.N., Yuan, L., Li, Q., Zhu, Y.J., Wang, M., and Sun, Y.G. (2020). Different neuronal populations mediate inflammatory pain analgesia by exogenous and endogenous opioids. Elife *9*, e55289.

Zhong, Q., Li, A., Jin, R., Zhang, D., Li, X., Jia, X., Ding, Z., Luo, P., Zhou, C., Jiang, C.*, et al.* (2021). High-definition imaging using line-illumination modulation microscopy. Nat Methods *18*, 309-315.

Zhou, L., Liu, M.Z., Li, Q., Deng, J., Mu, D., and Sun, Y.G. (2017). Organization of Functional Long-Range Circuits Controlling the Activity of Serotonergic Neurons in the Dorsal Raphe Nucleus. Cell Rep *20*, 1991-1993.
